# Photothermal CO_2_ Hydrogenation to Multicarbon Products: Mechanisms, Catalyst Design, and Scale Demonstration

**DOI:** 10.1007/s40820-026-02351-0

**Published:** 2026-11-12

**Authors:** Yanlin Liao, Ying Luo, Zheng Wang, Qiang Wang, Hong He

**Affiliations:** 1 Laboratory of Atmospheric Environment and Pollution Control, Research Center for Eco-Environmental Sciences, Chinese Academy of Sciences, Beijing, 100085 People’s Republic of China; 2 College of Environmental Science and Engineering, Beijing Forestry University, Beijing, 100083 People’s Republic of China; 3 College of Resource and Environment, University of Chinese Academy of Sciences, Beijing, 101408 People’s Republic of China; 4 State Key Laboratory of Advanced Environmental Technology, Institute of Urban Environment, Chinese Academy of Sciences, Xiamen, 361021 People’s Republic of China

**Keywords:** Photothermal catalysis, CO_2_ hydrogenation, C−C coupling, C_2+_ products, Scale demonstration

## Abstract

Photothermal CO_2_ hydrogenation to multicarbon products presents a promising route to mitigate global climate change while simultaneously generating valuable C_2+_ products for industrial use. However, realizing high activity and selectivity toward multicarbon products remains a significant challenge, primarily due to the intricate nature of reactant activation and the inherently sluggish kinetics of C−C coupling. This review elucidates the fundamental principles of photothermal catalysis, with a particular focus on the synergistic interplay between light and thermal energy. In addition, the activation pathways of CO_2_ to C_1_ intermediates and the subsequent mechanisms of C−C bond formation leading to multicarbon products are systematically examined. On this basis, catalyst design and modification strategies are categorized to enhance reactant activation and facilitate efficient C−C coupling. Furthermore, recent progress in pilot-scale demonstrations is highlighted to showcase the potential for scalable and sustainable CO_2_ hydrogenation technologies. By addressing the current challenges and outlining future research directions, this review aims to accelerate the development of efficient photothermal processes for CO_2_ valorization.

## Highlights

- Photothermal synergistic mechanism and reaction pathways from CO_2_ to C_2+_ products are systematically elucidated, providing a mechanistic basis for multicarbon product synthesis.
- Photothermal catalyst modification strategies are comprehensively summarized from a pathway-oriented perspective to enhance reactant activation and promote C−C coupling toward C_2+_ products.
- Recent photothermal scale demonstrations are highlighted, underscoring the practical potential of scalable and sustainable CO_2_ hydrogenation technologies.

## Graphical abstract

**Figure:**
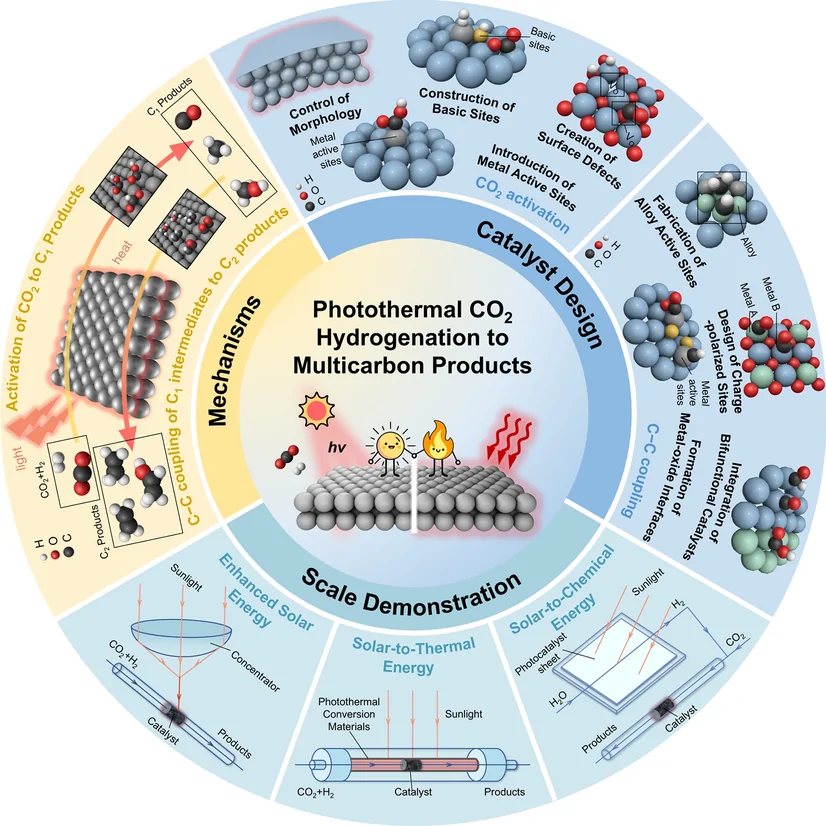

## Introduction

**Fig. 1:**
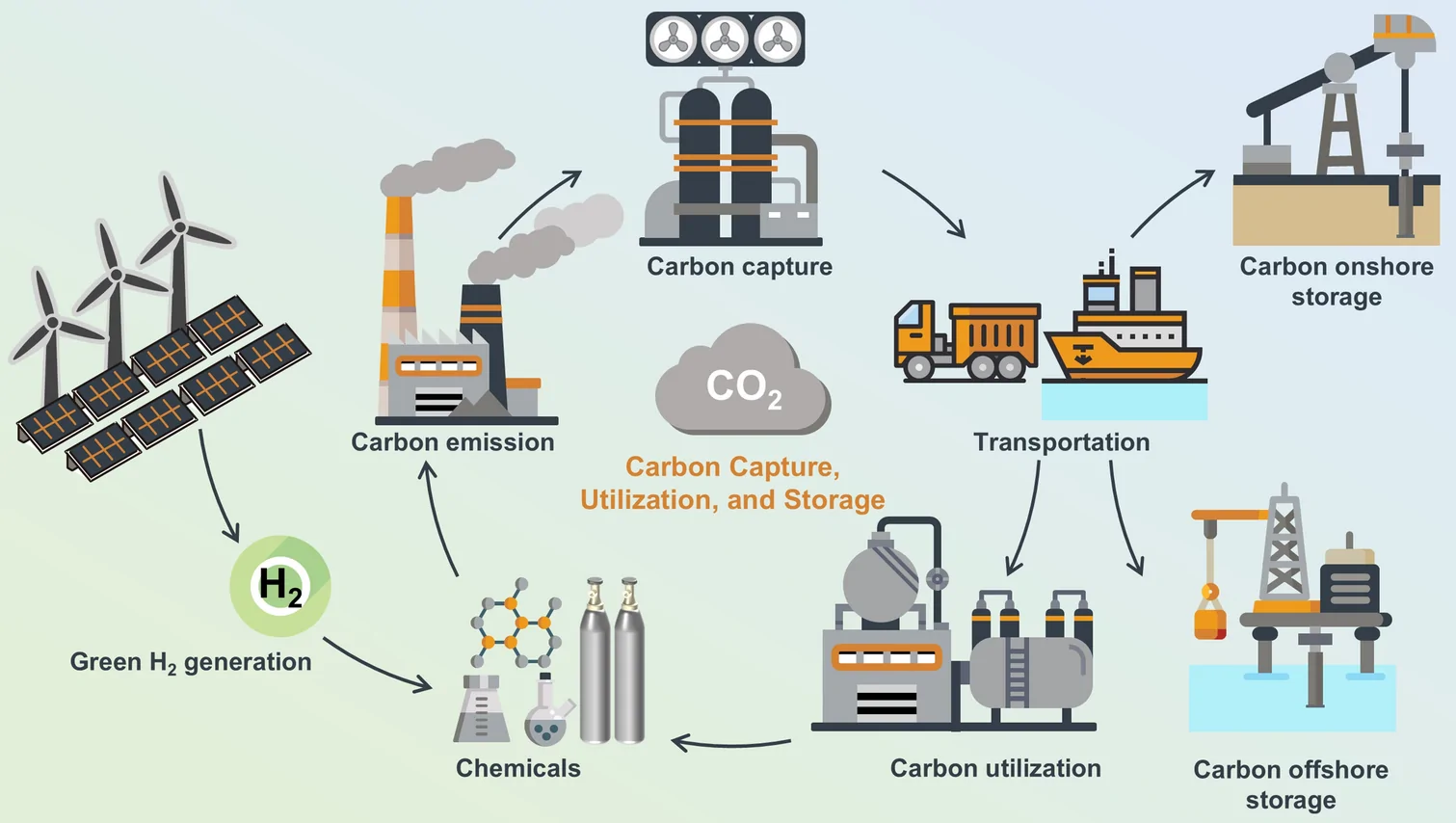
Conceptual figure exemplifying the major steps of CCUS

Over the past several decades, excessive fossil fuel consumption has resulted in CO_2_ emissions exceeding 30 billion tons annually, with profound impacts on human living conditions and global climate [1, 2]. Mitigating these emissions is therefore essential for curbing global warming and advancing toward a sustainable future [3, 4]. Among various mitigation strategies, carbon capture, utilization, and storage (CCUS) has attracted considerable attention owing to its comprehensive emission reduction potential and techno-economic feasibility. As illustrated in Fig. 1, CCUS involves CO_2_ capture from point sources, followed by either long-term geological storage or conversion into valuable products [5]. While storage-focused solutions provide an immediate way of emission control, they contribute minimally to resource valorization and economic sustainability. In contrast, CO_2_ utilization seeks to transform CO_2_ from environmental waste into a chemical feedstock, thereby enabling the production of value-added chemicals and supporting circular carbon economy [6, 7].

**Fig. 2:**
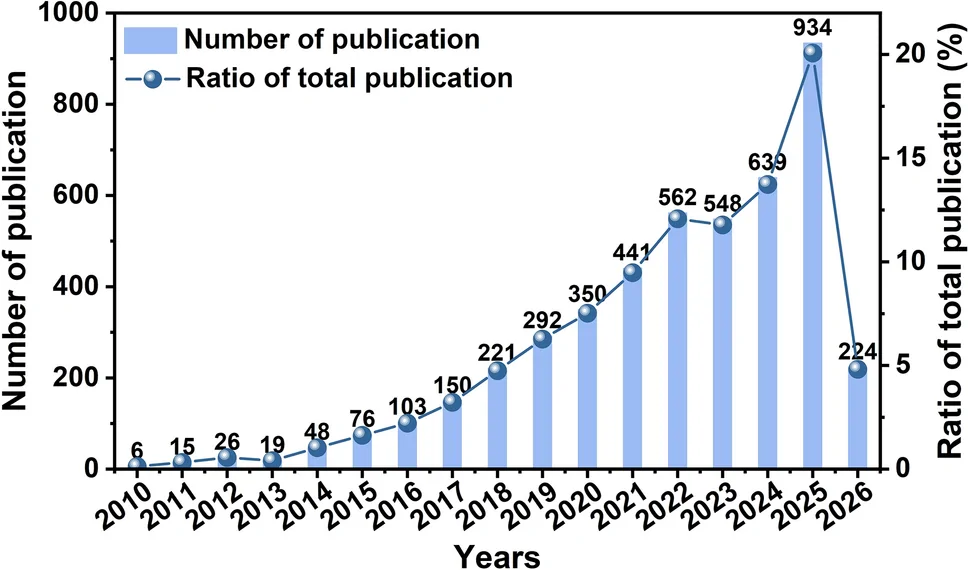
Recent publications and their respective ratios of total publications related to photothermal CO_2_ hydrogenation (Data for the graphs were obtained from the Web of Science Core Collection)

However, practical deployment of CO_2_ utilization technologies remains challenged by intrinsic thermodynamic stability of CO_2_ and energy-intensive nature of conventional catalytic processes [8–13]. To address these challenges, recent research has increasingly focused on catalytic systems driven by multiple physical fields [14–19]. Within this context, photothermal CO_2_ hydrogenation has garnered significant attention due to its low cost, high solar energy absorption, and independence from fossil fuel consumption (Fig. 2) [20, 21]. The underlying mechanism of photothermal CO_2_ hydrogenation combines photo-induced and thermally driven catalytic pathways [22]. The resulting synergy significantly enhances CO_2_ hydrogenation efficiency, positioning photothermal catalysis as a promising strategy for sustainable carbon conversion [23, 24].

Within the spectrum of products derived from photothermal CO_2_ hydrogenation, multicarbon compounds are of industrial importance due to their dual roles as energy-dense fuels and essential feedstocks for chemical synthesis [25, 26]. As a result, the efficient conversion of CO_2_ to C_2+_ products has become a key research priority. Although significant progress has been made in photothermal CO_2_ hydrogenation [27, 28], the selective formation of C_2+_ products remains challenging due to the inherently sluggish kinetics of C−C coupling [29, 30].

Although photothermal CO_2_ hydrogenation has been reviewed from the perspectives of material design, photothermal conversion, and reactor development, systematic discussions centered on multicarbon product formation remain relatively limited. In this review, we organize recent progress according to the key reaction sequence from CO_2_ activation and C_1_ intermediate formation to C–C coupling and C_2+_ product generation, with particular emphasis on photothermal synergy. This reaction-oriented framework enables the comparison of different coupling mechanisms, the evaluation of photothermal roles in pathway regulation, and the clarification of catalyst design principles for selective C_2+_ synthesis. Following this logic, the fundamental principles of photothermal catalysis, CO_2_ activation pathways leading to C_1_ intermediates, and subsequent C−C coupling mechanisms for C_2+_ product formation are systematically elucidated. On this basis, catalyst design and modification strategies are summarized to enhance reactant activation and promote C−C bond formation. In addition, recent progress in scale demonstrations and their feasibility is discussed to support the development of scalable and sustainable CO_2_ hydrogenation technologies. Finally, by identifying existing challenges and outlining future research directions, this review seeks to accelerate the development of efficient pathways for CO_2_ valorization.

## Fundamental Principles of Photothermal CO_2_ Hydrogenation

Photothermal catalysis leverages photonic energy, thermal energy, and their synergistic effects, offering an innovative strategy to overcome the kinetic constraints and thermodynamic limitations of conventional CO_2_ conversion reactions.

### Role of Light in Photothermal Catalysis

**Fig. 3:**
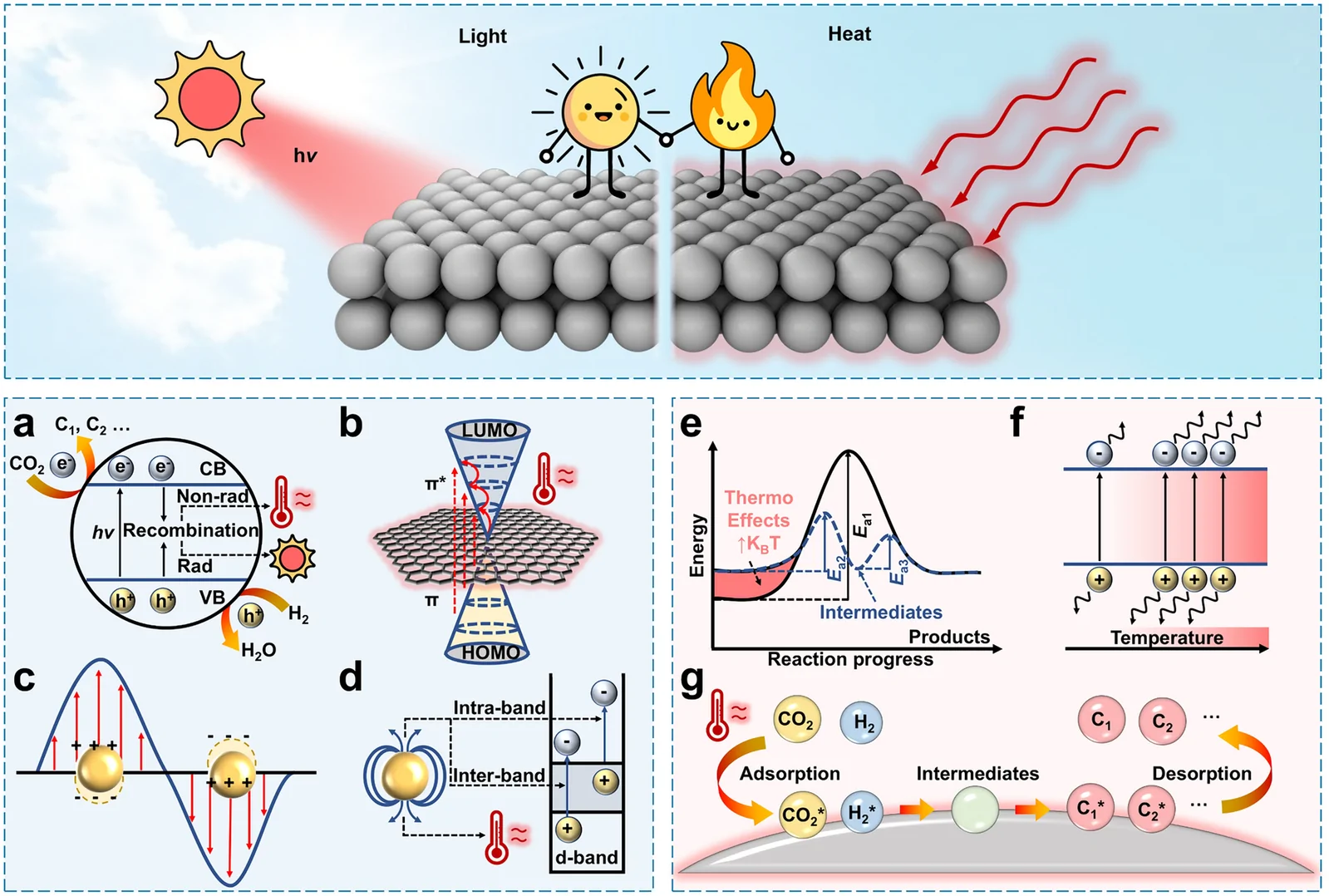
Role of light and heat in photothermal catalysis. **a** Light effect on semiconductors. **b** Molecular thermal vibration on carbon-based materials. Reproduced with permission from Ref. [39], Copyright 2024 John Wiley and Sons. **c** Dynamics of excited plasmonic nanoparticles. Reproduced with permission from Ref. [42], Copyright 2019 American Chemical Society.** d** Hot carrier generation induced by LSPR effect. Reproduced with permission from Ref. [47], Copyright 2022 The Royal Society of Chemistry. **e** Thermochemical contributions to energy path of endothermic reactions. Reproduced with permission from Ref. [24], Copyright 2019 The Royal Society of Chemistry.** f** Thermochemical contributions to charge carrier migration. **g** Mass transfer processes

The role of light in photothermal catalysis originates from intrinsic photocatalytic properties of semiconductors, whose tunable bandgap structures can efficiently generate charge carriers [31–33]. Specifically, as illustrated in Fig. 3a, when semiconductors absorb photons with energies equal to or greater than their bandgap, electrons in valence band are excited to conduction band, generating electron–hole pairs. These photogenerated carriers subsequently migrate to the surface of the semiconductor, where they participate in redox reactions with adsorbed CO_2_* and H_2_*.

In addition to the direct photoresponse of semiconductor materials, light can also induce photothermal conversion that facilitates catalytic reactions. In semiconductors, nonradiative relaxation represents the dominant photothermal conversion pathway [34, 35]. Upon illumination, electron–hole pairs undergo nonradiative recombination, where photoenergy transfers into lattice vibrations through electron–phonon coupling, resulting in localized heating [36]. For carbon-based materials, photothermal conversion proceeds through molecular vibrational excitation [37]. When incident photons possess energies matching electronic transition threshold, electrons are promoted from ground state to excited state [38]. Subsequently, via vibronic coupling, excited electrons undergo nonradiative relaxation back to the ground state, releasing excess energy as heat (Fig. 3b) [39, 40].

Noble metal nanostructures exhibit exceptional photothermal performance due to localized surface plasmon resonance (LSPR) [41]. Upon irradiation, conduction electrons in metal nanoparticles oscillate collectively to form surface plasmons. When the incident light frequency matches the resonance frequency, this collective oscillation is strongly amplified, significantly enhancing light absorption efficiency within the plasmonic metal system (Fig. 3c) [42, 43]. LSPR also intensifies local electric field at metal surface, promoting the generation of energetic charge carriers that facilitate reactant activation [44]. When this field enhancement peaks, part of the energy is released via radiative relaxation, while the majority dissipates through nonradiative processes. These nonradiative events generate energetic hot electrons, which may be injected into the conduction band of adjacent semiconductors or directly participate in surface redox reactions. Simultaneously, a portion of the absorbed energy is converted into heat, raising local reaction temperature and thereby enhancing catalytic kinetics (Fig. 3d).

### Role of Heat in Photothermal Catalysis

From a kinetic perspective, localized temperature field in photothermal systems plays a crucial role in regulating reaction rate. According to Arrhenius equation, an increase in temperature reduces the negative exponent, thereby enhancing the rate constant and accelerating elementary reaction steps. At molecular level, thermal activation enhances translational motion, thereby increasing both collision frequency and energy transfer efficiency during molecular collisions.

Furthermore, the influence of temperature can be interpreted from the perspective of the Boltzmann distribution. As temperature increases, the population of molecules occupying higher energy states rises due to slower decay of exponential energy distribution. This shift results in a substantially larger fraction of energetic species capable of initiating bond dissociation and overcoming activation energy barriers, thereby accelerating photothermal catalytic processes (Fig. 3e) [24].

Beyond accelerating intrinsic reaction kinetics, thermal effects also contribute to improved mass transport. In photothermal catalysis, localized heating can enhance charge carrier mobility and interfacial charge transfer efficiency (Fig. 3f) [45, 46], while in thermal-driven catalytic processes, it promotes reactant adsorption, intermediate migration, and product desorption, collectively leading to more efficient catalytic turnover (Fig. 3g) [47, 48].

### Strengthening Mechanism and Evaluation Criteria of Photothermal Synergy

The essence of photothermal synergy lies in the coupling of photonic and thermal effects. In principle, light irradiation can generate excited carriers and modulate surface electronic structures, while the accompanying thermal effect accelerates elementary reaction steps, intermediate diffusion, and mass transfer. The coupling of these two effects may enhance CO_2_ hydrogenation through three main pathways: promoting charge transport, lowering reaction barriers, and regulating reaction routes. However, enhanced activity under illumination does not necessarily indicate true photothermal synergy, because light may also simply serve as a heat source. Therefore, the discussion of photothermal synergy should not only describe the improved catalytic performance under light and heat, but also provide criteria to distinguish thermal-dominated effects, photochemical effects, and photothermal synergy.

Temperature-matched light/dark comparison is the basic criterion for this distinction. Comparing illuminated reactions with dark reactions only at the same furnace temperature is insufficient, because light irradiation may create local temperature gradients on the catalyst surface. If illumination changes the reaction rate, product selectivity, apparent activation energy, intermediate evolution, or electronic structure under temperature-matched conditions, non-thermal photochemical effects, or true photothermal synergy should be considered [49]. For example, to distinguish non-thermal photon effects from localized heating, direct and indirect illumination experiments were performed using a Ti_2_O_3_ overlayer, which provided comparable thermal input while blocking direct photon access to the catalyst surface. Under direct illumination, CO_2_ conversion showed a nearly linear dependence on light intensity in the range of 2.8–4.0 W cm^−2^, indicating a significant non-thermal photon contribution. By contrast, indirect illumination led to a nonlinear conversion trend dominated by thermal effects. Under comparable conditions, CO_2_ conversion under direct illumination was approximately 1.3 times that under indirect illumination, accompanied by an approximately 5% increase in C_2+_ product selectivity. Moreover, as the light intensity increased from 2.8 to 4.8 W cm^−2^, the conversion difference between direct and indirect illumination decreased from 36.94% to 20.96%, suggesting that the relative contribution of non-thermal effects declined at higher irradiance because of enhanced thermal accumulation [50]. On this basis, kinetic analysis, operando spectroscopy, electronic structure characterization, and carrier dynamics measurements are required to further identify the specific contribution of light and heat.

Within this framework, photothermal synergy first optimizes charge transport and proton transfer. In photothermal catalysis, light irradiation generates electron–hole pairs, while thermal energy promotes charge carrier separation and migration, thereby enhancing redox activity. Such carrier-related contributions are commonly supported by electrochemical characterizations, including time-resolved photoluminescence, transient photocurrent measurements, and electrochemical impedance spectroscopy, which provide information on carrier lifetime, separation efficiency, recombination behavior, and interfacial charge transfer resistance [51]. Meanwhile, thermal effects can also influence carrier dynamics. Elevated temperatures significantly reduce migration impedance of photogenerated carriers in materials such as TiO_2_, ZnO, and CeO_2_, thus facilitating more efficient charge transport [52–54]. However, excessive thermal input may also increase the electron–hole recombination rate, which can be mitigated through rational structural engineering of catalysts. LSPR provides an alternative route to enhance electron and proton transport under photothermal conditions [55, 56]. In Ru/SrTiO_3_ catalysts, LSPR-induced hot electrons and localized heating synergistically accelerated H_2_ dissociation, promoting their migration and spillover to the metal–support interface, thereby facilitating the hydrogenation of CO_2_ to CH_4_ [57].

Electronic structure characterization is also essential for identifying carrier-mediated photothermal synergy, especially when CO_2_ activation is proposed to proceed through surface redox cycles. In such cases, light irradiation not only increases local temperature but also induces electronic reconstruction and charge redistribution, whereas heat accelerates surface reaction kinetics. In situ X-ray photoelectron spectroscopy (XPS) and electron paramagnetic resonance (EPR) can provide such evidence by tracking electronic structure evolution under working conditions. For example, in Ru/LaFeO_3_-mediated photothermal dry reforming, in situ XPS and EPR revealed light-induced charge redistribution and oxygen vacancy cycling under illumination [58]. The thermal effect further accelerated the turnover of this redox cycle, thereby promoting CO_2_ activation and sustaining surface redox reactions beyond simple light-induced heating [59].

Second, photothermal synergy can reduce catalytic energy barriers. In this system, photoinduced carriers contribute redox capability, while thermal energy helps to overcome kinetic constraints. Apparent activation energies derived from Arrhenius analysis are frequently employed to quantify this effect under thermal and photothermal conditions [60]. Photothermal synergy can lower the apparent activation energy by approximately 10%−20%, primarily due to the direct involvement of photogenerated carriers, which introduce alternative reaction pathways [44, 54]. Furthermore, light excitation can modify the energy landscape by altering the adsorption behavior of key intermediates. In Pt/Al_2_O_3_ system, light irradiation induced CO migration from Pt step sites to terrace sites, weakening CO–Pt interactions and lowering the CO desorption barrier from 1.43 to 1.24 eV, thereby facilitating CO_2_ hydrogenation [61].

Finally, photothermal synergy enables regulation of reaction pathways and product selectivity. Photoexcitation can modulate electronic structure of catalyst surface, thereby altering the reaction mechanism and enabling control over product distribution and selectivity. In situ or operando vibrational spectroscopy is especially useful for distinguishing simple thermal acceleration from light-induced pathway regulation. When illumination changes the dominant intermediate, promotes the formation of a specific reactive species, or suppresses a competing route under comparable thermal conditions, pathway-level photothermal contribution can be inferred. Specifically, it was reported that photoexcitation can increase the electron density at metal sites, shifting CO_2_ activation pathway from thermally driven HCOO* intermediates to photothermally promoted COOH* species, which lowered activation barrier and enhanced hydrogenation activity [62]. Theoretical insights further suggested that temperature variation can alter the rate-determining step and associated energy barrier in C_1_ product formation, thereby influencing product selectivity. While higher temperatures can enhance the thermodynamic favorability of C_1_ product formation across different surface sites, an optimal temperature likely exists to maximize selectivity [36].

Overall, true photothermal synergy should not be assigned solely on the basis of enhanced catalytic activity under illumination. Its reliable identification requires surface temperature-matched light/dark comparisons, carrier dynamics measurements, electronic structure characterization, apparent activation energy analysis, and operando vibrational spectroscopy. These criteria are essential for distinguishing thermal-dominated enhancement from photothermal synergy.

## Reaction Pathway of Photothermal CO_2_ Hydrogenation to C_2_ Products

Understanding the reaction pathway of photothermal CO_2_ hydrogenation is essential for rational design of catalysts toward multicarbon products. The conversion of CO_2_ to C_2+_ products proceeds through a cascade reaction network involving CO_2_ activation, C_1_ intermediate formation, and subsequent C–C bond construction. In this network, the first stage involves CO_2_ activation and its conversion into C_1_ intermediates, including CO*, CH_x_*, and CH_3_OH-related species. The second stage involves C–C bond formation from these C_1_ intermediates, which directly governs the formation and selectivity of C_2+_ products.

### Reaction Pathway from CO_2_ to C_1_ Products

CO_2_ activation to C_1_ intermediates is the prerequisite for subsequent C–C coupling. Depending on the initial activation mode and hydrogenation sequence, CO_2_ can be converted into CO*, CH_x_*, and CH_3_OH-related species through direct C=O cleavage, COOH*-mediated pathways, or HCOO*-mediated pathways. These C_1_ species provide the upstream reactive intermediates for multicarbon product formation.

#### ***CO***_***2***_*** Hydrogenation to CO***

As the simplest C_1_ product of CO_2_ hydrogenation, CO plays a central intermediate role in downstream C−C coupling pathways. Owing to its endothermic nature $(\Delta_{R} H_{298K}^{0} = + 41.2 \,\,kJ\,\, mol^{ - 1} )$, CO formation is particularly sensitive to localized thermal effects, making it well suited for photothermal catalysis. Table 1 summarizes recently reported catalysts for photothermal CO_2_ hydrogenation to CO.

**Table 1:** Recent developments in photothermal CO_2_ hydrogenation to CO

Catalyst	Reactor type	P (bar)	Light	GHSV^a^ (mL g^−1^ h^−1^)	H_2_/CO_2_ ratio	T (°C)	CO sel.^b^ (%)	CO rate (mmol g^−1^ h^−1^)	Ref.^c^
Bi_2_O_3−x_	batch	N.R.^d^	LED lamp (0.011 W cm^−2^)	–	3:1	26	100	~0.01	[69]
In_2_O_3−x_(OH)_y_	batch	1	300 W Xe lamp (1 W cm^−2^)	–	1:1	150	N.R.	~0.4	[70]
In_2_O_3−x_	batch	1	300 W Xe lamp (0.1 W cm^−2^)	–	1:1	340	~100	103.2	[71]
Ni/LaInO_3_	batch	1	300 W Xe lamp (3 W cm^−2^)	–	4:1	280	~100	26.3	[67]
Pt/Al_2_O_3_	batch	N.R.	LED lamp (0.71 W cm^−2^)	–	6:1	400	~80	~4.8	[61]
PtRh/GaN/Si	batch	1	300 W Xe lamp (5.3 W cm^−2^)	–	12:1	350	98.5	11700	[46]
Co@CoN&C	batch	0.55	300 W Xe lamp	–	1:1	518	91.1	132	[72]
Co@C	batch	1	300 W Xe lamp (3 W cm^−2^)	–	1:1	380	92.6	536.7	[20]
FeO−CeO_2_	batch	1.8	300 W Xe lamp (2.2 W cm^−2^)	–	4:1	419	99.9	19.6	[73]
Cs/In_2_O_3_	flow	1	300 W Xe lamp	15,000	4:1	230	100	28	[74]
Pd/TiO_2_	flow	1	300 W Xe lamp (2.5 W cm^−2^)	24,000	4:1	250	86	7.2	[75]
Rh/TiO_2_	flow	1	300 W Xe lamp (2.7 W cm^−2^)	24,000	4:1	250	100	20.6	[76]
Ga−Cu/CeO_2_	flow	1	300 W Xe lamp (1.95 W cm^−2^)	12,000	1:1	280	~100	111.2	[77]
Cu_2_Zn_1_Al_0.5_Ce_5_Zr_0.5_O_x_	flow	1	Solar light source (0.1 W cm^−2^)	1,200	1:1	500	100	37.4	[68]
PI/A/S/S−Ni	flow	1	300 W Xe lamp (0.8 W cm^−2^)	1,622	5:1	230	87.3	516.9	[78]
Ni_3_N	flow	1	300 W Xe lamp (2.7 W cm^−2^)	2,310,000	1:10	199	99	1212	[79]
Mo_2_N/MoO_2−x_	flow	1	300 W Xe lamp (0.1 W cm^−2^)	240,000	1:1	250	~100	355	[80]
K^+^−Co−C	flow	1	300 W Xe lamp (2.8 W cm^−2^)	600,000	1:1	450	>95	758	[81]
Co_15_HAP	flow	1	300 W Xe lamp (0.108 W cm^−2^)	15,600	1:1	400	83	62	[82]

^a^GHSV, gas hourly space velocity. ^b^sel., selectivity. ^c^Ref., reference. ^d^N.R., not reported

**Fig. 4:**
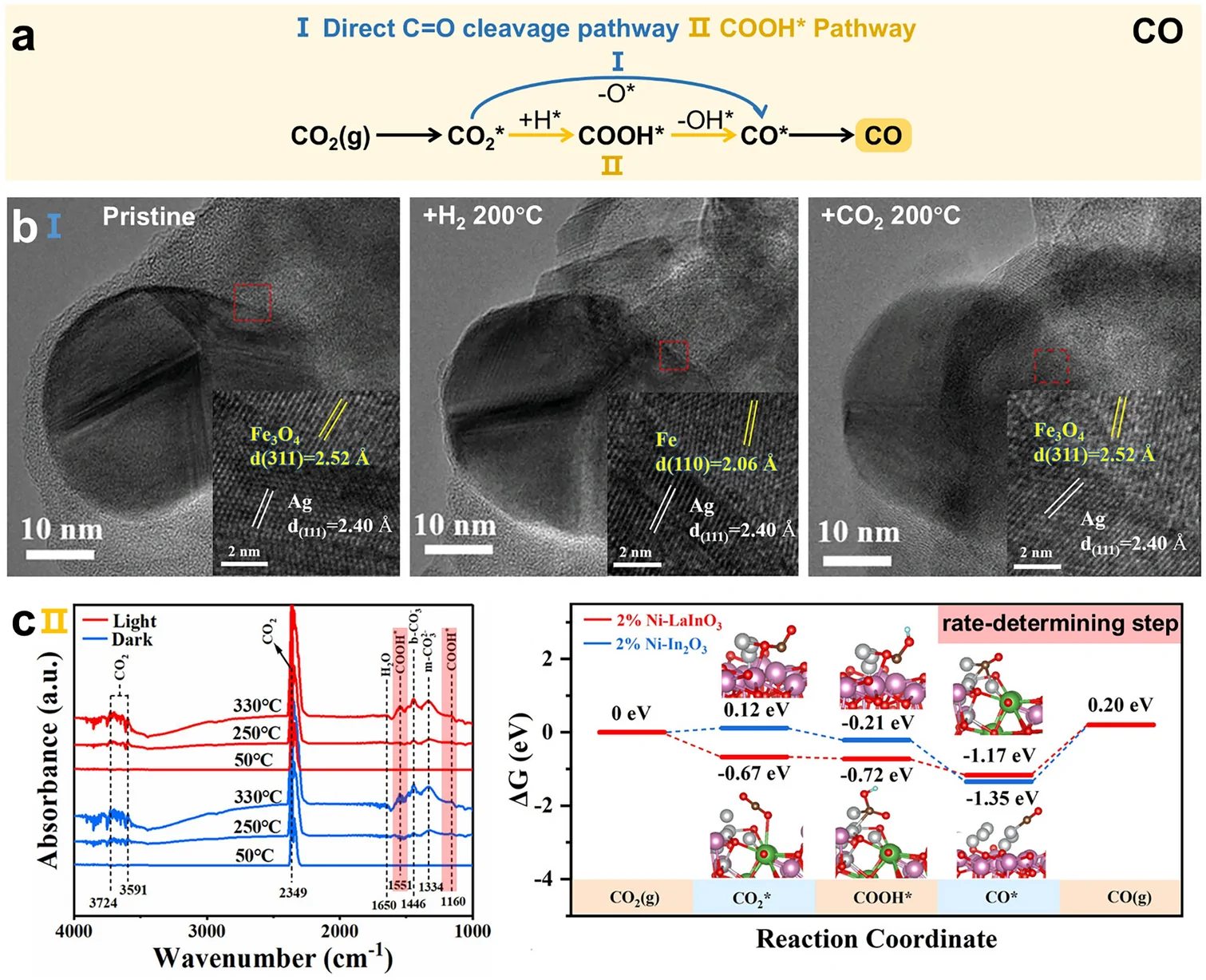
Reaction pathways for photothermal CO_2_ hydrogenation to CO. **a** Possible reaction pathways. **b** Direct C=O cleavage pathway revealed by in situ transmission electron microscopy (TEM) and density functional theory (DFT) calculations. Reproduced with permission from Ref. [64], Copyright 2025 John Wiley and Sons. **c** COOH* pathway revealed by in situ DRIFTS. Reproduced with permission from Ref. [67], Copyright 2023 John Wiley and Sons

CO can be produced through direct cleavage of C=O bond, with the release of an oxygen atom at oxygenophilic sites to form adsorbed CO* intermediate (Fig. 4a). Consequently, catalysts capable of reversibly binding oxygen species are particularly effective for this pathway by preventing surface oxygen accumulation and sustaining active-site availability [63]. In this context, metal–metal oxide redox cycling contributes to efficient CO formation, as H-induced reduction of Fe_3_O_4_ to metallic Fe enables direct C=O dissociation of CO_2_ to form CO, followed by reoxidation to Fe_3_O_4_ (Fig. 4b) [64]. Beyond metallic centers, dynamically generated oxygen vacancies further promote C=O bond cleavage by capturing terminal oxygen atoms from CO_2_ under photothermal conditions [65, 66].

In addition to oxygen abstraction-driven C=O bond cleavage, photothermal CO_2_ hydrogenation to CO frequently follows a COOH*-mediated reaction pathway, which can be directly observed through in situ diffuse reflectance infrared Fourier transform spectroscopy (DRIFTS) (Fig. 4c) [67]. Moreover, it was observed that the accumulation rate of COOH* intermediates on catalyst surfaces under concentrated light illumination is significantly higher than under dark thermal conditions, underscoring the advantages of photothermal coupling [46, 68].

#### ***CO***_***2***_*** Hydrogenation to CH***_***4***_

**Fig. 5:**
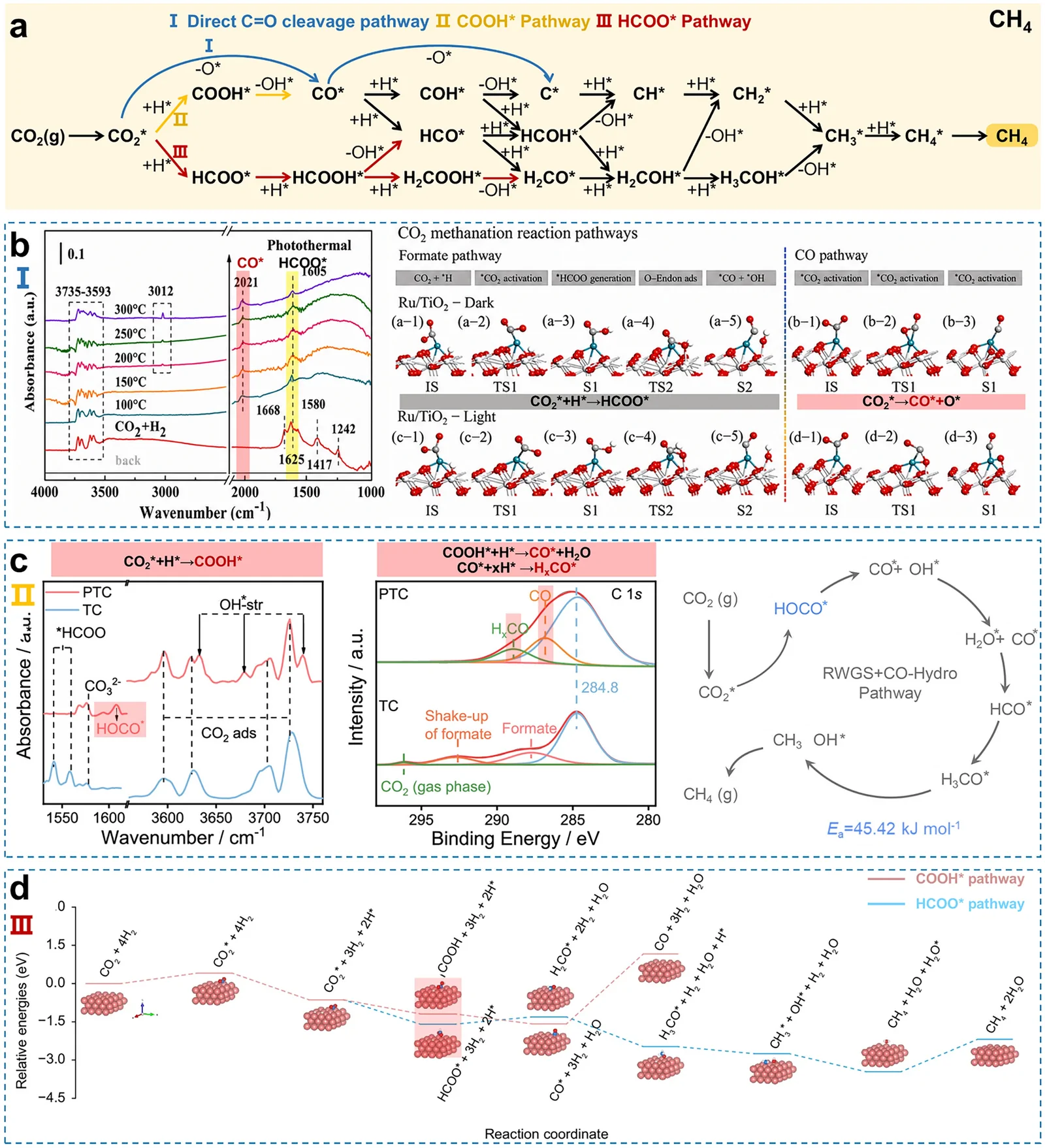
Reaction pathways for photothermal CO_2_ hydrogenation to CH_4_. **a** Possible reaction pathways. **b** Direct C=O cleavage pathway revealed by in situ DRIFTS and DFT calculations. Reproduced with permission from Ref. [84], Copyright 2022 Elsevier. **c** COOH* pathway revealed by in situ DRIFTS and in situ AP-XPS. Reproduced with permission from Ref. [62], Copyright 2021 American Chemical Society. **d** HCOO* pathway revealed by DFT calculations. Reproduced with permission from Ref. [92], Copyright 2024 Springer Nature

Unlike CO formation, photothermal CO_2_ hydrogenation to CH_4_ requires cleavage of both C=O bonds through multiple hydrogenation steps, rendering the process mechanistically more complex. Despite the existence of multiple pathways, CH_4_ formation commonly proceeds through surface CH_x_* intermediates as the main species (Fig. 5a). Table 2 summarizes recent advances in photothermal CO_2_ hydrogenation to CH_4_.

**Table 2:** Recent developments in photothermal CO_2_ hydrogenation to CH_4_

Catalyst	Reactor type	P (bar)	Light	GHSV (mL g^−1^ h^−1^)	H_2_/CO_2_ ratio	T (°C)	CH_4_ sel. (%)	CH_4_ rate (mmol g^−1^ h^−1^)	Ref.
In_2_O_3_@In	batch	1.2	300 W Xe lamp (0.26 W cm^−2^)	–	4:1	250	>99	1.04	[93]
Ni@TiO_2_	batch	N.R.	300 W Xe lamp (2.5 W cm^−2^)	–	4:1	420	81.8	286.5	[86]
MnO−Co	batch	1	300 W Xe lamp (1 W cm^−2^)	–	4:1	150	86.1	5.19	[62]
Co_3_O_4_	batch	15	Xe lamp	–	4:1	140	97.7	21.1	[94]
Ru/TiO_2_	flow	1	300 W Xe lamp	11,000	4:1	300	87	N.R.	[84]
RuCo/TiO_2_	flow	1	300 W Xe lamp (1.9 W cm^−2^)	24,000	4:1	250	94.6	191	[89]
Ru/H_x_MoO_3−y_	flow	1	Vis-NIR light (0.75 W cm^−2^)	4000	1:1	140	100	20.8	[95]
Ru/MnCo_2_O_4_	flow	1	300 W Xe lamp (1.25 W cm^−2^)	24,000	4:1	230	96	66.3	[96]
Ru/MnO/Mn_3_O_4_	flow	1	300 W Xe lamp (2.5 W cm^−2^)	40,000	4:1	200	99.5	166.7	[97]
Ru/Mg−CeO_2_	flow	1	300 W Xe lamp	200,000	4:1	400	93.1	469	[98]
Co/CeO_2_	flow	1	300 W Xe lamp	12,000	3:1	200	97.3	885.3	[99]
Ni/CeO_2_	flow	1	300 W Xe lamp (~6 W cm^−2^)	32,000	4:1	~270	>99	9.12	[100]
Ni/TiO_2_	flow	1	300 W Xe lamp (15.3 W cm^−2^)	150,000	4:1	283	96.9	41.5	[101]
50Ni/ZrO_2_	flow	1	300 W Xe lamp (2.9 W cm^−2^)	150,000	4:1	394	95.4	583.3	[102]
MnNiZrRuCe HEMG	flow	1	300 W Xe lamp (1.14 W cm^−2^)	90,000	4:1	300	~100	290	[103]
Ir−CoO/Al_2_O_3_	flow	1	300 W Xe lamp (2 W cm^−2^)	24,000	4:1	250	92	128.9	[91]
Ir@UiO-66	flow	1	300 W Xe lamp (2.3 W cm^−2^)	24,000	4:1	250	~95	19.9	[104]
Pt/LaCoO_3_	flow	1	300 W Xe lamp (1.2 W cm^−2^)	24,000	4:1	250	87	119.8	[105]

In direct C=O bond cleavage pathway, one C=O bond of the adsorbed CO_2_ molecule is cleaved to form CO*, followed by the cleavage of the second C=O bond under hydrogenation. Dalmon et al. (1978) first observed infrared bands corresponding to CO* vibrations during the chemisorption of CO_2_ on a Ni/SiO_2_ catalyst. Based on these observations, they proposed a pathway involving CO_2_ (gas) → CO* + O* [83]. In photothermal catalytic systems, CO* and HCOO* species were detected on a Ru/TiO_2_ catalyst, suggesting that the CO_2_-to-CH_4_ conversion can follow direct C=O bond cleavage pathway (Fig. 5b) [84].

In COOH* pathway, chemisorbed CO_2_* reacts with dissociated H* to form a COOH* intermediate, which subsequently decomposes into CO* and H_2_O. Depending on the hydrogenation position during the second C=O bond breakage, this pathway can be further divided into the COOH*-HCO* and COOH*-COH* routes [85]. Among them, the COOH*-HCO* pathway has been more extensively investigated experimentally [62, 86], whereas the COOH*-COH* pathway is more frequently proposed in theoretical studies [87, 88]. In a MnO−Co catalytic system, COOH* species were identified via in situ DRIFTS, while hydrogenation products such as CO* and H_x_CO* were detected by in situ ambient pressure X-ray photoelectron spectroscopy (AP-XPS) C 1*s* spectra. These experimental findings provide compelling evidence for the COOH*-HCO* pathway in photothermal CO_2_-to-CH_4_ conversion (Fig. 5c) [62].

Moreover, CH_4_ can be produced through HCOO* pathway, where CO_2_ is initially hydrogenated at the carbon site to form an HCOO* intermediate [89, 90]. HCOO* then undergoes further hydrogenation at the oxygen site to produce HCOOH*. HCOOH* may either hydrogenate to H_2_COOH* and subsequently dehydrate to form CH_2_* [91], or hydrogenate to H_3_COOH* and dehydrate to yield CH_3_*, ultimately leading to CH_4_ formation. After experimentally observing the HCOO* intermediate, Jia Zhu et al. employed density functional theory (DFT) calculations to investigate the CO_2_ methanation pathway over Ru@m-Ti_3_C_2_T_x_. HCOO* pathway exhibited more favorable thermodynamics than the COOH* pathway, ultimately confirming its dominance in photothermal CO_2_ hydrogenation to CH_4_ (Fig. 5d) [92].

#### ***CO***_***2***_*** Hydrogenation to CH***_***3***_***OH***

CH_3_OH is a simple saturated monohydric alcohol with a high hydrogen storage capacity per unit mass and volume, making it an ideal target product for photothermal CO_2_ hydrogenation [106]. However, its formation is intrinsically challenging because it requires the cleavage of one C=O bond while retaining the other. Table 3 summarizes recent progress in photothermal CO_2_ hydrogenation to CH_3_OH. Additionally, CH_3_OH also serves as a key intermediate in the reaction network for CO_2_ hydrogenation to C_2+_ products, providing mechanistic insights for improving C_2+_ product yields [107].

**Table 3:** Recent developments in photothermal CO_2_ hydrogenation to CH_3_OH

Catalyst	Reactor type	P (bar)	Light	GHSV (mL g^−1^ h^−1^)	H_2_/CO_2_ ratio	T (°C)	CH_3_OH sel. (%)	CH_3_OH rate (μmol g^−1^ h^−1^)	Ref.
CoPc/CNT	batch	40	1000 W Xe lamp (2.0 W cm^−2^)	–	3:1	~245	~99	2400	[109]
In_2_O_3_	batch	6	Xe lamp (1.2 W cm^−2^)	–	3:1	200	61	1436	[115]
Au/In_2_O_3_	batch	25	300 W Xe lamp (0.1054 W cm^−2^)	–	3:1	250	~56	320	[116]
Pd_3_Cu@UiO-66	batch	12.5	300 W Xe lamp	–	3:1	200	88	340	[117]
CaCu_3_Ti_4_O_12_	flow	1	130 W Xe lamp	4800	3:1	250	17	14.1	[118]
Cu/ZnO	flow	1	Xe lamp (0.58 W cm^−2^)	60000	3:1	220	~13	2.1	[119]
In_2_O_3−x_(OH)_y_	flow	1	130 W Xe lamp	32000	3:1	250	53	63.7	[110]
H_z_In_2_O_3−x_(OH)_y_	flow	1	300 W Xe lamp (0.6 W cm^−2^)	N.R.	1:1	250	18.9	7.2	[120]
rh-In_2_O_3−x_(OH)_y_	flow	1	130 W Xe lamp	24000	3:1	270	~13	180	[113]
Bi_x_In_2−x_O_3_	flow	1	300 W Xe lamp (2 W cm^−2^)	24000	3:1	230	14.7	158	[114]
Ru/In_2_O_3_	flow	1	300 W Xe lamp	120000	3:1	250	18	280.4	[108]

**Fig. 6:**
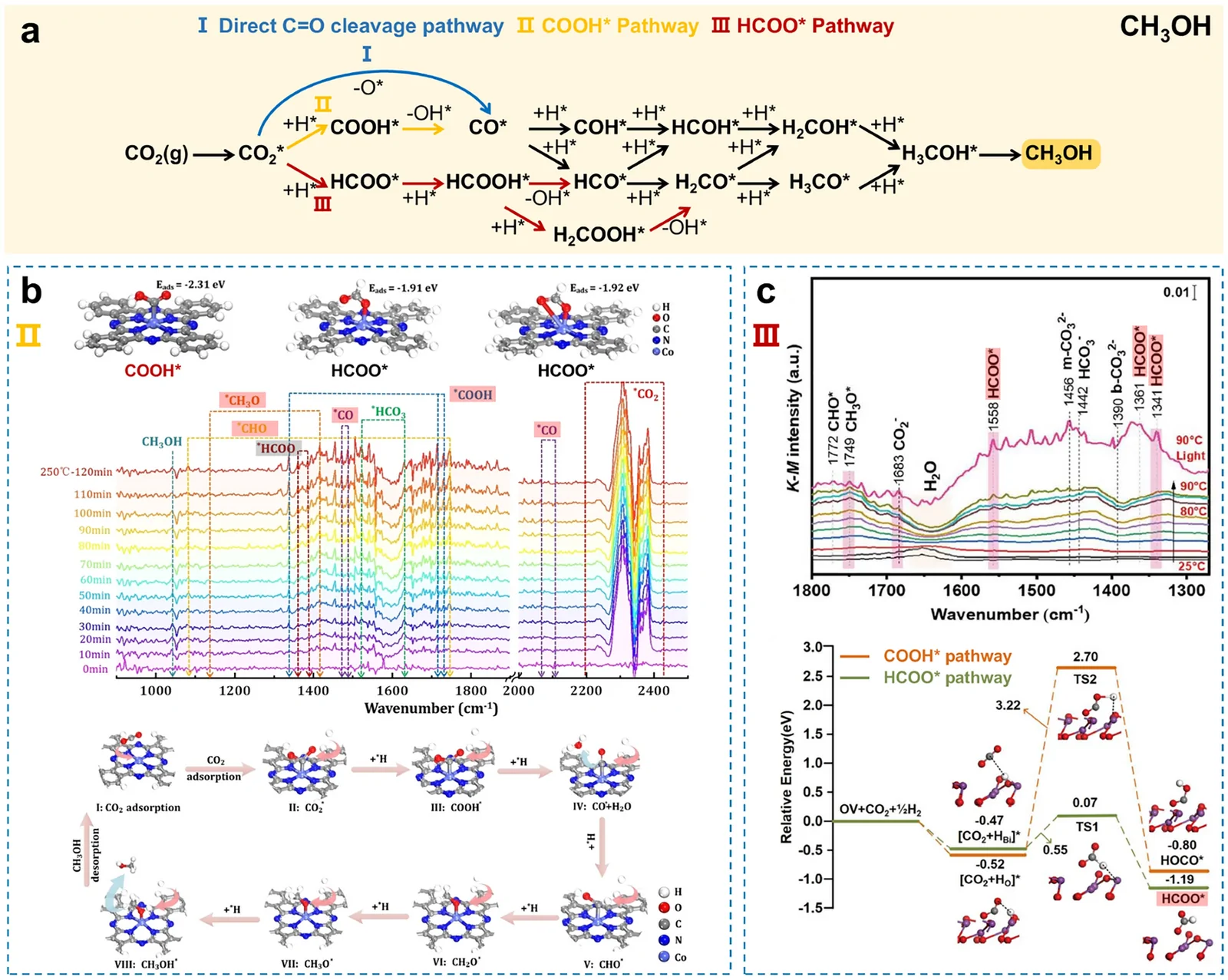
Reaction pathways for photothermal CO_2_ hydrogenation to CH_3_OH. **a** Possible reaction pathways. **b** COOH* pathway revealed by in situ DRIFTS and DFT calculations. Reproduced with permission from Ref. [109], Copyright 2024 John Wiley and Sons. **c** HCOO* pathway revealed by in situ DRIFTS and DFT calculations. Reproduced with permission from Ref. [112], Copyright 2025 Springer Nature

To date, CH_3_OH synthesis via photothermal CO_2_ hydrogenation typically proceeds through two primary intermediates: COOH* and HCOO* (Fig. 6a) [108]. The COOH* pathway involves two consecutive steps: (1) CO_2_ is hydrogenated to form COOH*, which is further reduced to CO*, and (2) CO* is subsequently hydrogenated to produce CH_3_OH [108, 109]. In contrast, in HCOO* pathway, CO_2_ is initially hydrogenated at the carbon site to form HCOO*, which is then converted into HCOOH*. This intermediate subsequently generates CH_3_O* and undergoes further hydrogenation to form CH_3_OH [110]. It has also been reported that HCOOH* can follow an alternative hydrogenation route to generate HCO*, which is further hydrogenated at the oxygen site to produce CH_3_OH [111].

In typical studies of photothermal CO_2_ hydrogenation to CH_3_OH, mechanistic pathways are often investigated by first applying in situ DRIFTS to detect surface intermediates, followed by DFT calculations to validate the proposed pathways. However, DRIFTS-based assignments of intermediates, including COOH*, HCOO*, and CH_3_O* species, should be treated cautiously because their vibrational bands may overlap with carbonate or bicarbonate spectator species, and short-lived intermediates may remain undetected. Therefore, DFT calculations and isotope-labeling experiments are often needed to distinguish dominant reaction routes from merely observable surface species. For example, on a cobalt phthalocyanine (CoPc)/carbon nanotube system, the introduction of CO_2_ and H_2_ led to the observation of stretching vibrations associated with COOH*, along with a weak absorption band attributed to HCOO*. Subsequent DFT calculations assessed the thermodynamic stability of adsorbed COOH* and HCOO*, revealing that HCOO* was thermodynamically less stable. Furthermore, the absence of hydrogenation intermediates derived from HCOO* in operando DRIFTS further evidenced COOH* pathway (Fig. 6b) [109]. HCOO* was first directly identified on Bi_2_WO_6_ nanosheets, and subsequent DFT calculations of CO_2_ adsorption and hydrogenation further confirmed the thermodynamic feasibility of HCOO* pathway (Fig. 6c) [112]. Moreover, both COOH* and HCOO* pathways can coexist in photothermal CO_2_ hydrogenation reactions, potentially working synergistically to enhance CH_3_OH production [113, 114].

### Reaction Pathway from C_1_ Intermediates to C_2_ Products

After C_1_ intermediates are generated, C–C bond formation becomes the decisive step for C_2+_ product synthesis. According to the dominant coupling species, C–C coupling in CO_2_ hydrogenation can be classified into CO*-mediated, CH_x_*-mediated, and CH_3_OH*-mediated routes. Table 4 summarizes recent progress in the photothermal hydrogenation of CO_2_ toward multicarbon products.

**Table 4:** Recent developments in photothermal CO_2_ hydrogenation to multicarbon products

Catalyst	Reactor type	P (bar)	Light	GHSV (mL g^−1^ h^−1^)	H_2_/CO_2_ ratio	T (°C)	C_2+_ rate (mmol g^−1^ h^−1^)	Sel. (%)			Ref.
								C_2+_	CO	CH_4_	
TiO_2_	batch	0.2	200 W Hg lamp (0.15 W cm^−2^)	–	1.5:1	120	~0.0041	3.1	44.7	52.2	[121]
NiFeO_x_	batch	2.7	1000 W Xe lamp (5 W cm^−2^)	–	5:1	300	~12.72	21.6	0.7	77.7	[122]
NiO_x_/Nb_2_O_5_	batch	1	N.R.	–	6:1	250	0.194	57.12	2.01	40.86	[123]
Na−Co@C	batch	2.8	1000 W Xe lamp (2.4 W cm^−2^)	–	5:1	235	4.12	39.7	0	62.7	[124]
MnO−Co	batch	1	300 W Xe lamp (1 W cm^−2^)	–	4:1	150	0.63	15.87	0	84.13	[62]
Co_7_Cu_1_Mn_1_O_x_	batch	1	300 W Xe lamp (0.234 W cm^−2^)	–	5:1	200	1.40	30.9	3.7	65.4	[125]
Fe/C	batch	1	300 W Xe lamp (2.05 W cm^−2^)	–	3:1	310	~0.82	15.2	59.8	25	[126]
Fe_3_C	batch	1	300 W Xe lamp (2.05 W cm^−2^)	–	2:1	310	0.89	11.4	25.3	63.4	[126]
K−Ru/Fe_3_O_4_	batch	1	300 W Xe lamp (2.05 W cm^−2^)	–	3:1	420	0.63	23.8	67.2	9	[127]
CoFe alloy/Al_2_O_3_	batch	1.8	300 W Xe lamp (5.2 W cm^−2^)	–	4:1	320	~0.42	35.3	5.0	59.8	[128]
Fe/FeO_x_/Al_2_O_3_−MgO	batch	1.8	300 W Xe lamp (1.88 W cm^−2^)	–	4:1	275	0.32	42.2	20.3	37.5	[129]
CoFe_2_O_4_/(Fe_1−x_Co_x_)_5_C	batch	3	300 W Xe lamp (1.7 W cm^−2^)	–	3:1	305	2.64	63.4	19.4	17.2	[130]
Fe_3_C/ZnO	flow	0	homemade light source (0.12 W cm^−2^)	2400	3:1	321	0.63	~25.80	~62.0	~12.20	[131]
K-Fe_10_Mn_1_	flow	20	300 W Xe lamp (4 W cm^−2^)	12000	4:1	250	4.80	59.6	24.1	16.3	[50]
CoFe alloy/Al_2_O_3_	flow	1.8	300 W Xe lamp (2.16 W cm^−2^)	9000	4:1	250	~0.26	21.01	45.95	33.04	[132]
NiFe alloy/Al_2_O_3_	flow	1.8	300 W Xe lamp (2.16 W cm^−2^)	9000	4:1	250	~0.74	0.96	1.24	97.79	[132]
NiCo alloy/Al_2_O_3_	flow	1.8	300 W Xe lamp (2.16 W cm^−2^)	9000	4:1	250	~0.52	0.29	0.79	99.44	[132]
CoFe/CoFe_2_O_4_	flow	1	300 W Xe lamp (2 W cm^−2^)	7895	4:1	300	1.10	29.8	50	21.2	[133]
Co−CoOx/MgAl_2_O_4_	flow	3	LED (1.2 W cm^−2^)	2400	3:1	325	1.16	35.2	24.3	40.5	[134]
Cu_0.65_/Co_0.35_Fe_2_	flow	1	LED (0.045 W cm^−2^)	14400	3:1	320	~1.6	31.8	56.9	11.3	[135]

#### CO* Coupling

**Fig. 7:**
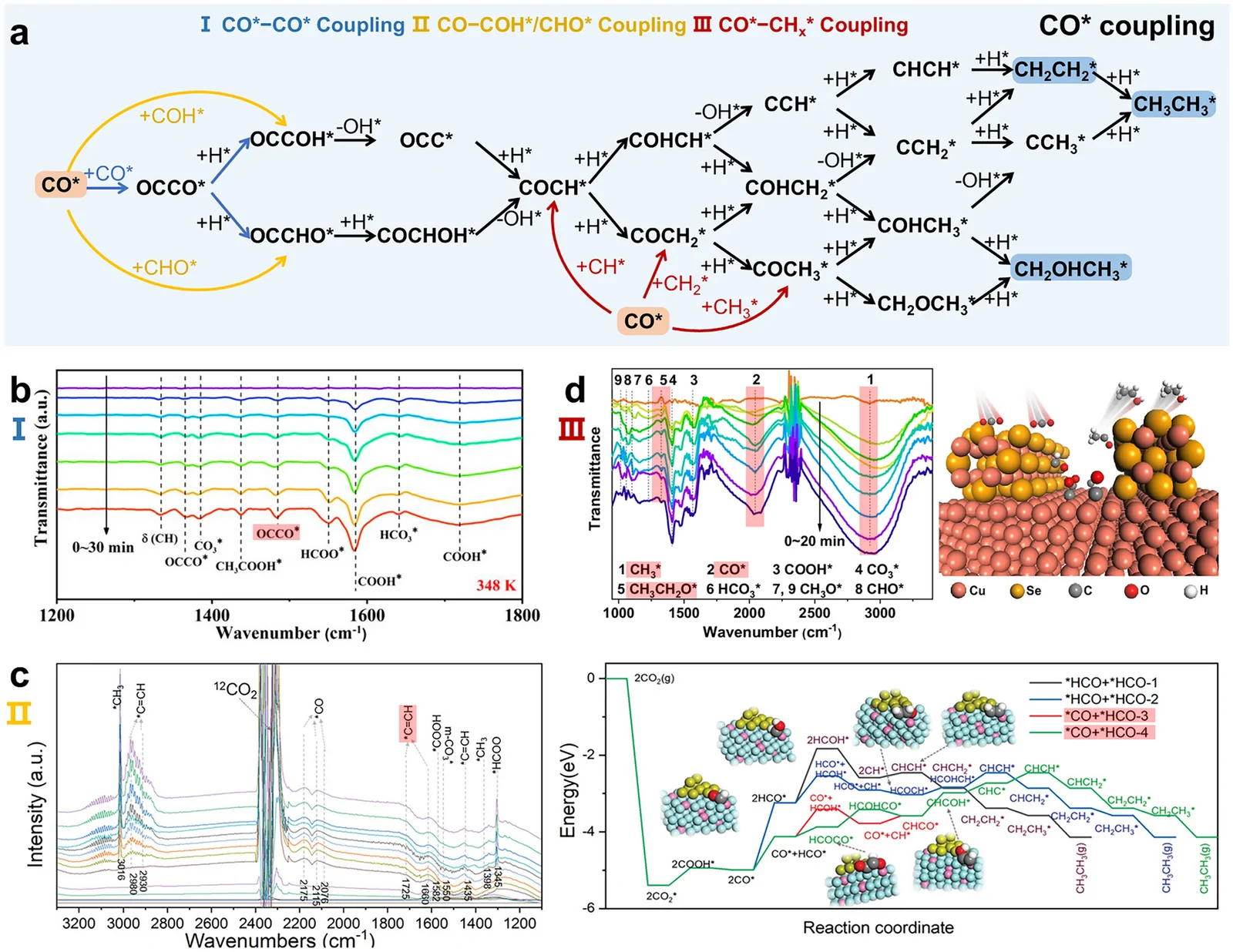
Reaction pathways for CO_2_ hydrogenation to C_2_ products via CO* coupling. **a** Possible reaction pathways via CO* coupling. **b** CO*−CO* pathway revealed by in situ DRIFTS. Reproduced with permission from Ref. [137], Copyright 2021 American Chemical Society. **c** CO*−HCO* pathway revealed by in situ DRIFTS and DFT calculations. Reproduced with permission from Ref. [135], Copyright 2024 John Wiley and Sons. **d** CO*−CH_3_* pathway revealed by in situ DRIFTS. Reproduced with permission from Ref. [140], Copyright 2024 Springer Nature

CO* coupling in photothermal CO_2_ hydrogenation to C_2_ products can be further classified into CO*−CO* coupling, CO*−COH*/HCO* coupling, and CO*−CH_x_* coupling (Fig. 7a).

In CO_2_ hydrogenation, the widely accepted C−C coupling mechanism begins with the reduction of CO_2_ to CO*, followed by the direct coupling of two CO* intermediates to form OCCO* species. This intermediate then undergoes successive hydrogenation and deoxygenation steps to yield C_2_ products. Under photothermal conditions, AuCu alloy-modified g-C_3_N_4_ system proposed a direct CO*−CO* coupling mechanism, where CO_2_ was initially reduced via COOH* pathway to CO*, and subsequently dimerized to form OCCO* [136]. Additionally, OCCO* intermediate was identified as vibrational peak during photothermal CO_2_ reduction over Zn−O−Ge tri-atomic sites, providing direct evidence for the CO*−CO* coupling route (Fig. 7b) [137].

However, some studies suggested that the formation of OCCO* is associated with a relatively high energy barrier [138], whereas coupling between CO* and hydrogenated species (e.g., HCO* or COH*) proceeds with lower activation energies, from an energy barrier of 1.52 eV (CO*+CO*) to 1.08 eV (CO*+CHO*) on Cu/TiO_2_ [139]. In a Cu-promoted CoFe alloy catalyst, C_1_ intermediates including HCO* and CO*, along with C_2_ intermediates, C=CH*, were detected during CO_2_ hydrogenation process. On this basis, mechanistic analysis suggested that the most thermodynamically favorable pathway involved the coupling of CO* and HCO* to form OCCHO*, followed by successive hydrogenation steps to generate COCH*, CHC*, and ultimately multicarbon alkenes and alkanes (Fig. 7c) [135].

Additionally, coupling between CO* and CH_x_* species, leading to the formation of COCH_x_*, represents another viable pathway for C_2_ product generation. It is reported that ethanol can be formed via CO*−CH_3_* coupling, based on the detection of CO*, CH_3_*, and CH_3_CH_2_O* intermediates (Fig. 7d) [140].

Overall, CO*-mediated coupling requires sufficient CO* coverage and suitable adsorption configurations to stabilize C–C coupling intermediates. Direct CO*–CO* coupling can be promoted on sites with asymmetric electronic structures, whereas coupling between CO* and partially hydrogenated species is often more favorable when the surface can balance CO* retention and hydrogenation. Photothermal catalysis may further facilitate this process by combining localized thermal activation, which promotes intermediate mobility and collision, with photoinduced electronic modulation, which optimizes adsorption configuration and charge distribution of CO*-derived species [137].

#### ***CH***_x_* Coupling

**Fig. 8:**
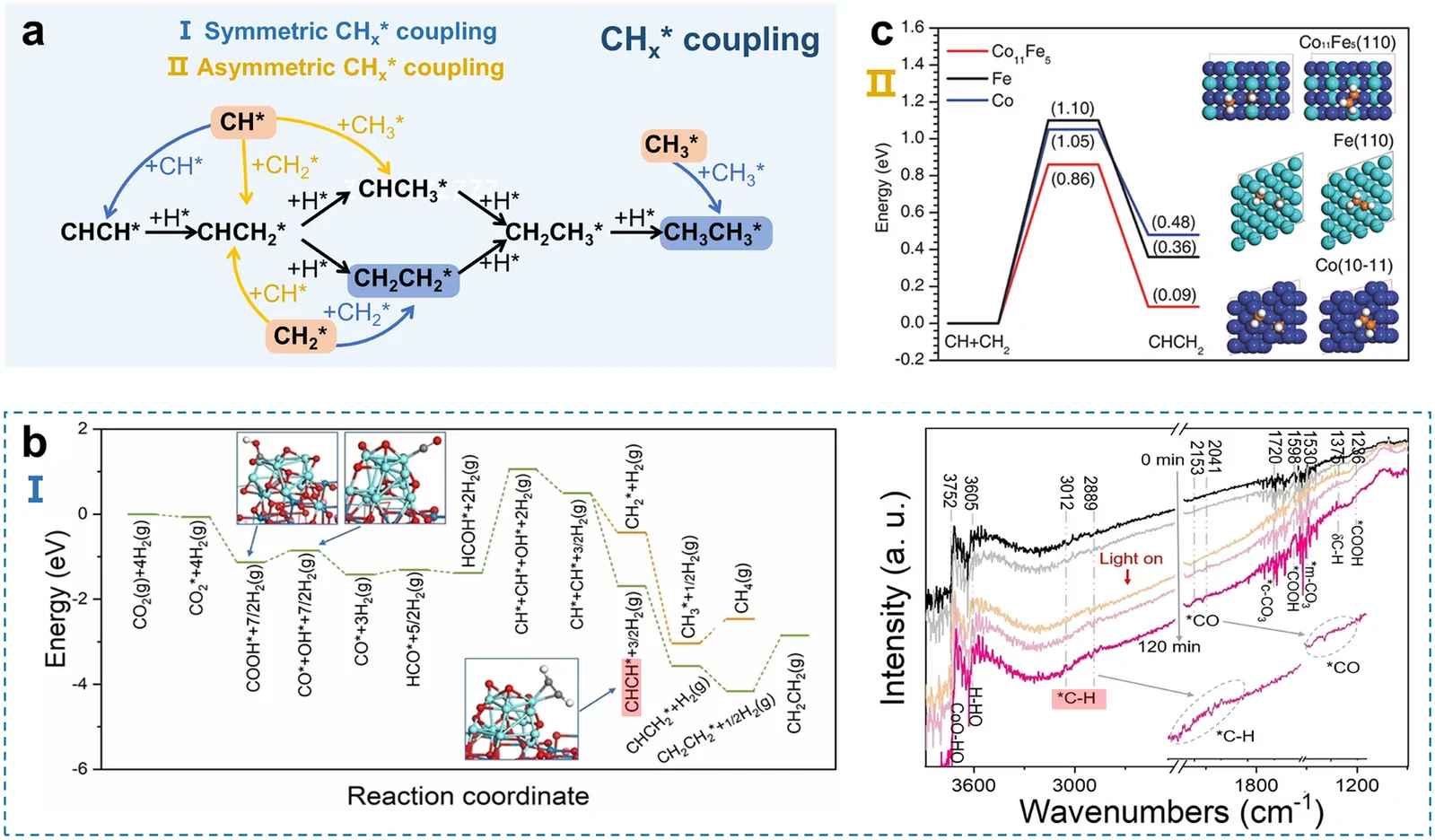
Reaction pathways for CO_2_ hydrogenation to C_2_ products via CH_x_* coupling. **a** Possible reaction pathways via CH_x_* coupling. **b** Symmetric CH*−CH* pathway revealed by in situ DRIFTS and DFT calculations. Reproduced with permission from Ref. [134], Copyright 2023 John Wiley and Sons. **c** Asymmetric CH*−CH_2_* pathway revealed by DFT calculations. Reproduced with permission from Ref. [128], Copyright 2017 John Wiley and Sons

In photothermal CO_2_ hydrogenation, C_2_H_4_ and C_2_H_6_ are typically formed via C−C coupling between CH_x_* intermediates (Fig. 8a). On a Co−CoO_x_/MgAl_2_O_4_ catalyst, CH* species can couple to form CHCH* during photothermal catalysis, which yield C_2_H_4_ and C_2_H_6_ (Fig. 8b) [134]. Similarly, in another study on photothermal CO_2_ hydrogenation to C_2_ products, CH_2_*−CH_2_* coupling was identified as the dominant route for carbon chain elongation [127]. In addition, CH_3_*−CH_3_* coupling has also been proposed as a principal pathway for C_2_H_6_ formation [123].

Beyond symmetric CH_x_* coupling, C−C bond formation can also occur through asymmetric coupling. Tierui Zhang et al. reported a CoFe alloy/Al_2_O_3_ catalyst, which exhibited high C_2_ selectivity in the CO_2_ hydrogenation process. According to DFT calculations, the most favorable C−C coupling pathway on the Co_11_Fe_5_(110) surface involves the coupling of CH* and CH_2_* with an adsorbed barrier of 0.86 eV (Fig. 8c) [128].

In summary, CH_x_*-mediated coupling requires a delicate balance between the formation of hydrogenated C_1_ intermediates and the suppression of excessive hydrogenation to CH_4_. Photothermal catalysis is particularly useful for this route because localized heating can accelerate the generation and migration of CH_x_* species, while photoinduced charge redistribution at alloy or metal–oxide interfaces can tune their hydrogenation degree and coupling tendency. Together with the CO*-mediated route discussed above, these pathways demonstrate that light-induced electronic modulation and localized thermal activation can jointly promote C_1_ intermediate formation and subsequent C–C coupling in photothermal CO_2_ hydrogenation. To clarify the mechanistic distinction among different coupling routes, Table 5 summarizes their reaction pathway, key intermediates, and representative catalysts.

**Table 5:** Reaction pathways from CO_2_ activation to C_1_ intermediates and C_2+_ products in photothermal CO_2_ hydrogenation

Reaction stage	Pathway	Key intermediates	Reported Products	Representative catalysts	Ref.
CO_2_ activation to C_1_ intermediates	Direct C=O cleavage	CO*, HCO*, COH*, CH_x_*	CO, CH_4_	AgK/Fe_3_O_4_, Ru/TiO_2_	[64, 84]
	COOH* pathway	CO*, HCO*, COH*, CH_3_OH*, CH_x_*	CO, CH_3_OH, CH_4_	Ni/LaInO_3_, Ru/In_2_O_3_, Ni/TiO_2_	[67, 86, 108]
	HCOO* pathway	HCOOH*, HCO*, H_2_COOH*, CH_3_OH*, CH_x_*	CH_3_OH, CH_4_	Ir-CoO/Al_2_O_3_, IrPd-In_2_O_3_	[91, 111]
C–C coupling to C_2+_ products	CO*-mediated coupling	CO*–CO*, CO*–HCO*, CO*–CHO*, CO*–CH_x_*	C_2_H_4_, C_2_H_6_, C_2_H_5_OH	Zn_2_GeO_4_, Cu/CoFe, Cu_2_Se-Cu_2_O	[135, 137, 140]
	CH_x_*-mediated coupling	CH_x_*–CH_x_*, CH*–CH_x_*, CH_2_*–CH_3_*	C_2_H_4_, C_2_H_6_	Co−CoO_x_/MgAl_2_O_4_, CoFe/Al_2_O_3_	[128, 134]

#### ***CH***_***3***_OH* Coupling

Unlike the CO*- and CH_x_*-mediated routes discussed above, CH_3_OH*-mediated C–C coupling has been primarily established in thermal catalysis. The CH_3_OH*-mediated pathway is considered an effective route for producing olefins and alkanes, as this pathway circumvents the limitations of the Anderson–Schulz–Flory (ASF) distribution and significantly enhances the selectivity toward higher hydrocarbons [141–143]. Although methanol can clearly be generated through COOH* or HCOO* pathways, subsequent C−C coupling of methanol to form olefins, alkanes, or even aromatics is highly complex. This complexity has sparked ongoing debate over direct versus indirect mechanisms, primarily due to the lack of direct spectroscopic evidence. Consequently, more than twenty reaction pathways have been proposed to account for the formation of these products. Specifically focusing on C_2_ product formation, some researchers have suggested that CH_3_OH* can couple with another CH_3_OH* intermediate via dehydration to form CH_3_OCH_3_*, which can then undergo hydrogenation and deoxygenation to generate CHCH_2_* [144, 145]. It has also been reported that CH_3_OH* can couple with COOH* to form HCOOCH_3_*, which subsequently undergoes hydrogenation to produce CHCH_2_*. This intermediate may then proceed through further hydrogenation and C−C coupling steps to yield aromatic compounds [146, 147]. Additionally, HCOO* intermediate has also been implicated in CH_3_OH-mediated C−C coupling reactions [148].

CH_3_OH*-mediated CO_2_ hydrogenation generally involves a tandem catalytic system. These systems employ bifunctional catalysts composed of metal oxides and zeolites, wherein the metal oxide component facilitates the hydrogenation of CO_2_ to methanol, which then diffuses into the zeolite phase for further conversion into multicarbon hydrocarbons. Owing to their well-defined pore structures and abundant Brønsted acid sites, zeolites serve as shape-selective catalysts and play a pivotal role in methanol coupling pathways during thermal CO_2_ hydrogenation to higher hydrocarbons [149]. However, incorporating zeolite catalysts into photothermal CO_2_ hydrogenation systems remains challenging due to two primary limitations. On the one hand, conventional zeolites, mainly composed of aluminosilicates, possess wide bandgaps and can only absorb ultraviolet light (λ < 400 nm) [150, 151]. As visible and infrared light account for the majority of solar irradiation, their poor absorption in these regions results in limited photothermal conversion efficiency, making it difficult to reach the temperatures required for efficient photothermal catalysis. On the other hand, photogenerated charge carriers must migrate to the catalyst surface to participate in redox reactions of photothermal catalysis. Previous studies have shown that shorter migration distances promote charge separation, while longer pathways increase the probability of recombination [152]. The extended pore networks in zeolites inherently prolong the migration distance, thereby exacerbating carrier recombination. Additionally, the high electrical resistivity of aluminosilicate zeolites further impedes the effective transport of photogenerated electrons and holes, intensifying recombination losses and diminishing photothermal catalytic performance [153, 154]. Thus, future research should focus on enhancing the photothermal responsiveness of zeolite-based catalysts and improving charge carrier dynamics to enable their broader use in photothermal catalytic systems.

## Modification Strategies for Photothermal CO_2_ Hydrogenation Catalysts

**Fig. 9:**
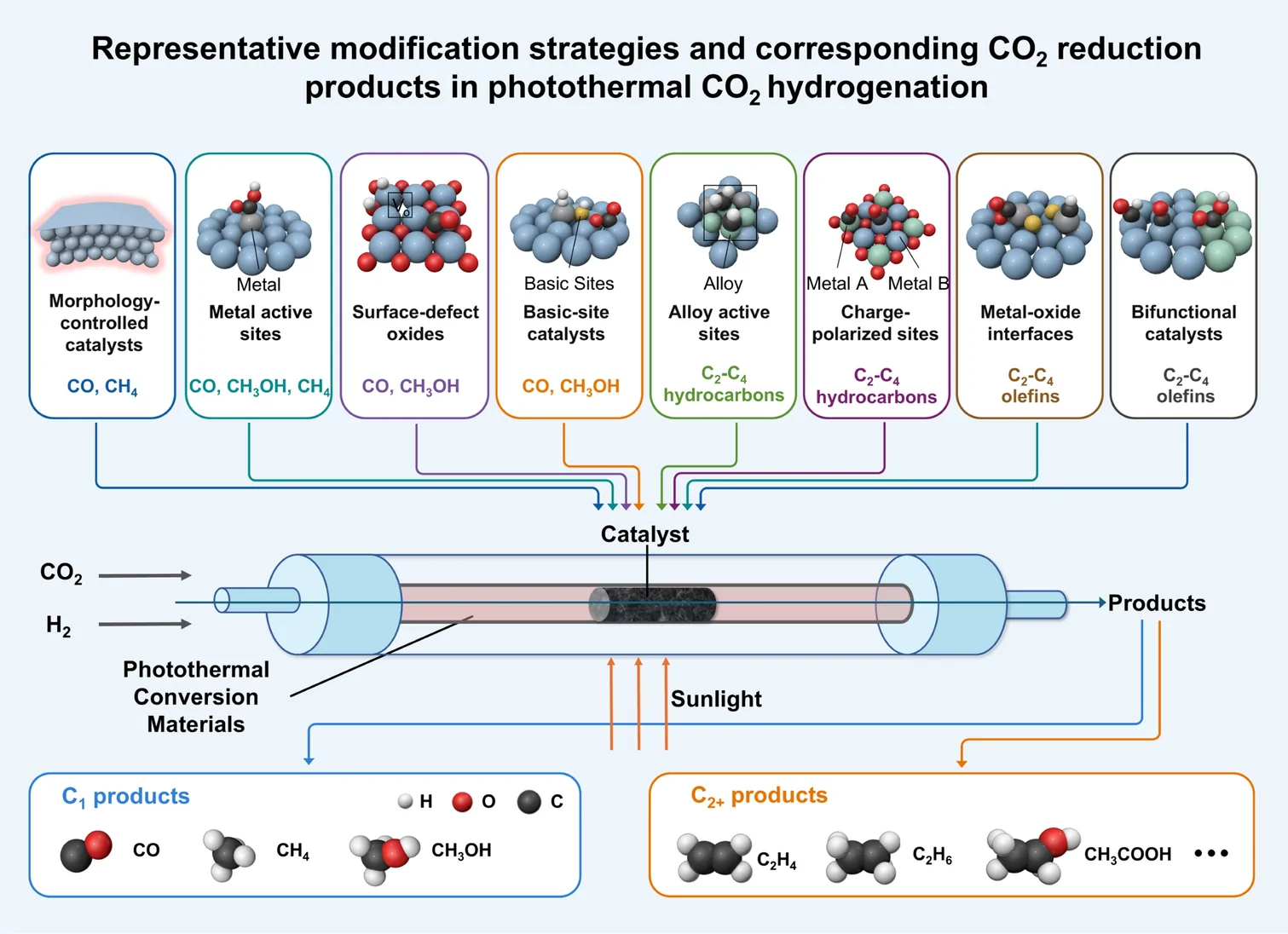
Representative modification strategies and corresponding CO_2_ reduction products in photothermal CO_2_ hydrogenation

Based on pathways discussed above, both product yield and selectivity toward C_2+_ hydrocarbons are predominantly governed by two key steps: CO_2_ activation to C_1_ intermediates and subsequent C−C coupling of these intermediates. Accordingly, we propose a series of modification strategies aimed at improving reaction performance by facilitating the activation of CO_2_ to C_1_ species and promoting efficient C−C coupling (Fig. 9).

### Facilitating CO_2_ Activation to C_1_ Products

#### Control of Morphology

In photothermal CO_2_ hydrogenation, catalyst morphology plays a pivotal role in determining both the photothermal conversion efficiency and the efficiency of reactant activation. By tailoring the surface properties and microstructure of the catalyst, it is possible to significantly enhance solar-to-thermal energy conversion as well as increase the number of accessible active sites. Such morphology optimization strategies offer new opportunities for realizing efficient CO_2_ hydrogenation into C_1_ products.

1. Photothermal conversion efficiency

**Fig. 10:**
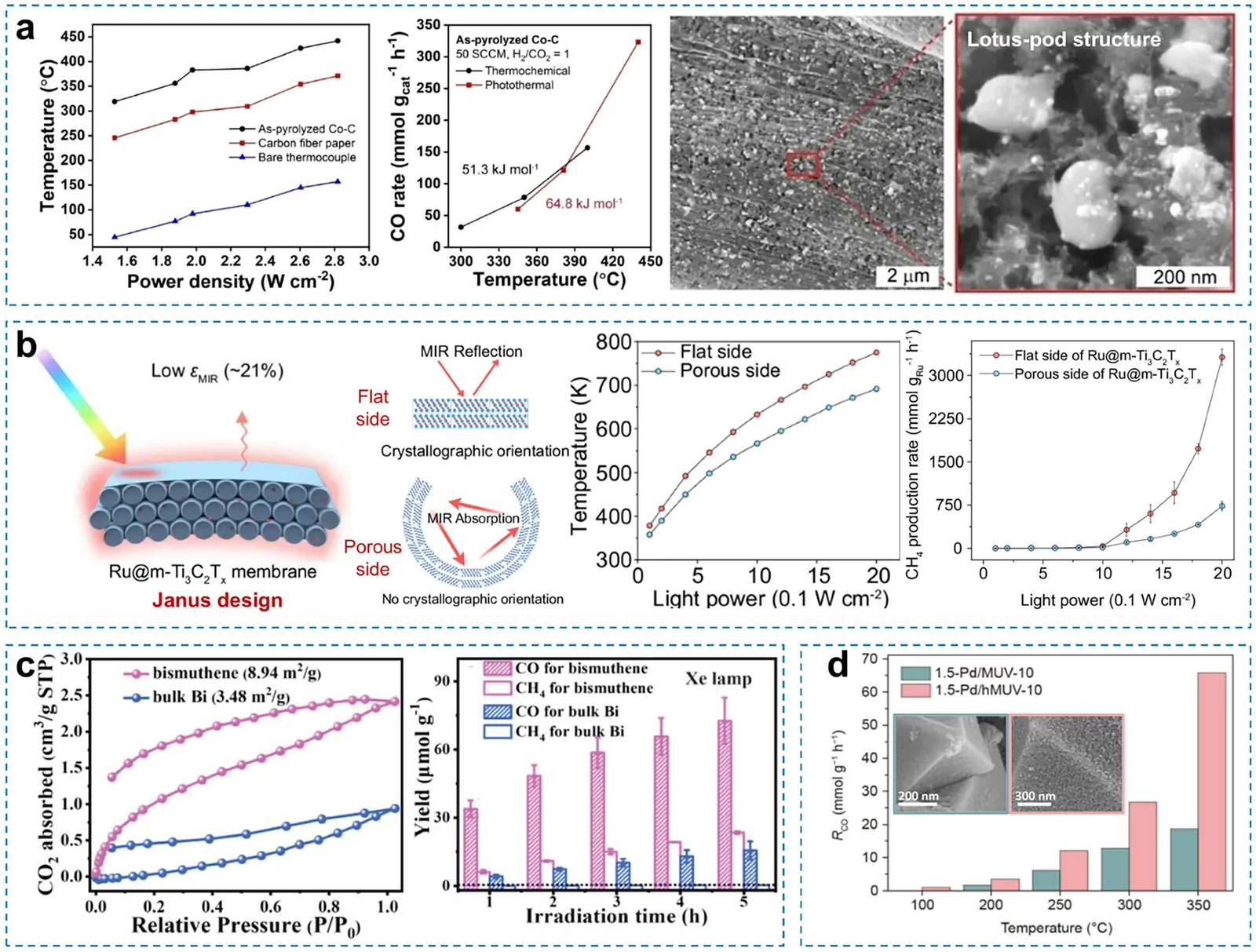
Effect of morphology control on facilitating the activation of CO_2_ to C_1_ products. **a** Light absorption enhancement by hierarchical porous-structured K^+^−Co−C. Reproduced with permission from Ref. [81], Copyright 2023 John Wiley and Sons. **b** Thermal radiation suppression by smooth side of Janus-structured Ru@m-Ti_3_C_2_T_x_. Reproduced with permission from Ref. [92], Copyright 2024 Springer Nature. **c** Specific surface area increases by bismuthene nanosheets. Reproduced with permission from Ref. [159], Copyright 2024 John Wiley and Sons. **d** Porous-structured construction by Pd/hMUV-10 with hierarchical mesopores. Reproduced with permission from Ref. [161], Copyright 2023 Springer Nature

To maximize photothermal conversion efficiency, catalysts should achieve strong solar light absorption while minimizing thermal radiation losses under illumination [155–157]. Morphological modifications have proven effective in boosting photothermal efficiency. Hierarchical porous structure of K^+^−Co−C can enhance photon trapping and scattering within the bulk, thereby increasing local temperature under light intensity and ultimately improving photothermal reaction performance (Fig. 10a) [81]. The unique hierarchical structures of nanocages enable Ru-loaded carbon nanocages achieved nearly 90% light absorption across the solar spectrum. Under 2.4 W cm^−2^ light irradiation, the catalyst exhibited a record CO production rate of 3.1 mol g_Ru_^−1^ h^−1^ in flow reactor, with selectivity exceeding 90% [158].

Beyond enhancing light absorption, morphological control also helps reduce thermal emissivity. In Janus-structured Ru@m-Ti_3_C_2_T_x_ materials for photothermal CO_2_ methanation, the smooth side exhibited a remarkably low mid-infrared emissivity (~21%), which effectively suppressed radiative heat loss and enabled localized heat accumulation, while the porous side facilitated reactant diffusion and product transport, together leading to enhanced CH_4_ yields (Fig. 10b) [92].

(2) Reactant activation capability

Catalyst morphology also plays a key role in reactant activation, as both surface and internal regions typically serve as primary sites for CO_2_ and H_2_ activation. Increasing the specific surface area is a widely used strategy to improve reactant adsorption and activation. Compared with bulk materials, nanostructured catalysts offer significantly higher surface areas and a greater number of active sites, thus enhancing reactant activation capability. Nanostructured bismuthene sheets exhibited a photothermal CO_2_ hydrogenation activity 4.6 times higher than that of commercial bulk bismuth (Fig. 10c) [159].

Porous materials are promising for photothermal CO_2_ hydrogenation because their tailored pore structures can simultaneously improve reactant adsorption, mass transport, and active-site dispersion [160]. For example, a Pd-loaded hierarchically porous titanium-based metal–organic framework (Pd/hMUV-10) achieved a CO yield of 65.9 mmol g^−1^ h^−1^ under photothermal conditions at 350 °C. Its performance was attributed to the etching-generated mesoporous structure, which enhanced CO_2_ adsorption and activation, and to the hierarchical pore network, which facilitated uniform Pd dispersion across both internal and external surfaces (Fig. 10d) [161].

In summary, morphology control mainly strengthens the physical basis of photothermal catalysis by improving light absorption, heat localization, reactant diffusion, and active-site exposure. Nevertheless, these effects primarily facilitate upstream CO_2_ activation and do not necessarily ensure selective product formation. Therefore, morphology optimization should be integrated with chemical active-site design to achieve more efficient and selective CO_2_ hydrogenation.

#### Construction of Basic Sites

Basic sites, including alkali metals, alkaline earth metals, and certain transition metal oxides, are widely introduced to enhance CO_2_ activation in photothermal hydrogenation. Owing to their intrinsic basicity, alkali and alkaline earth promoters strengthen the interaction between CO_2_ and catalyst surface, thereby facilitating CO_2_ activation. By contrast, transition metal oxides generally promote reactant activation through electronic modulation of catalyst surface.

1. Alkali metals

**Fig. 11:**
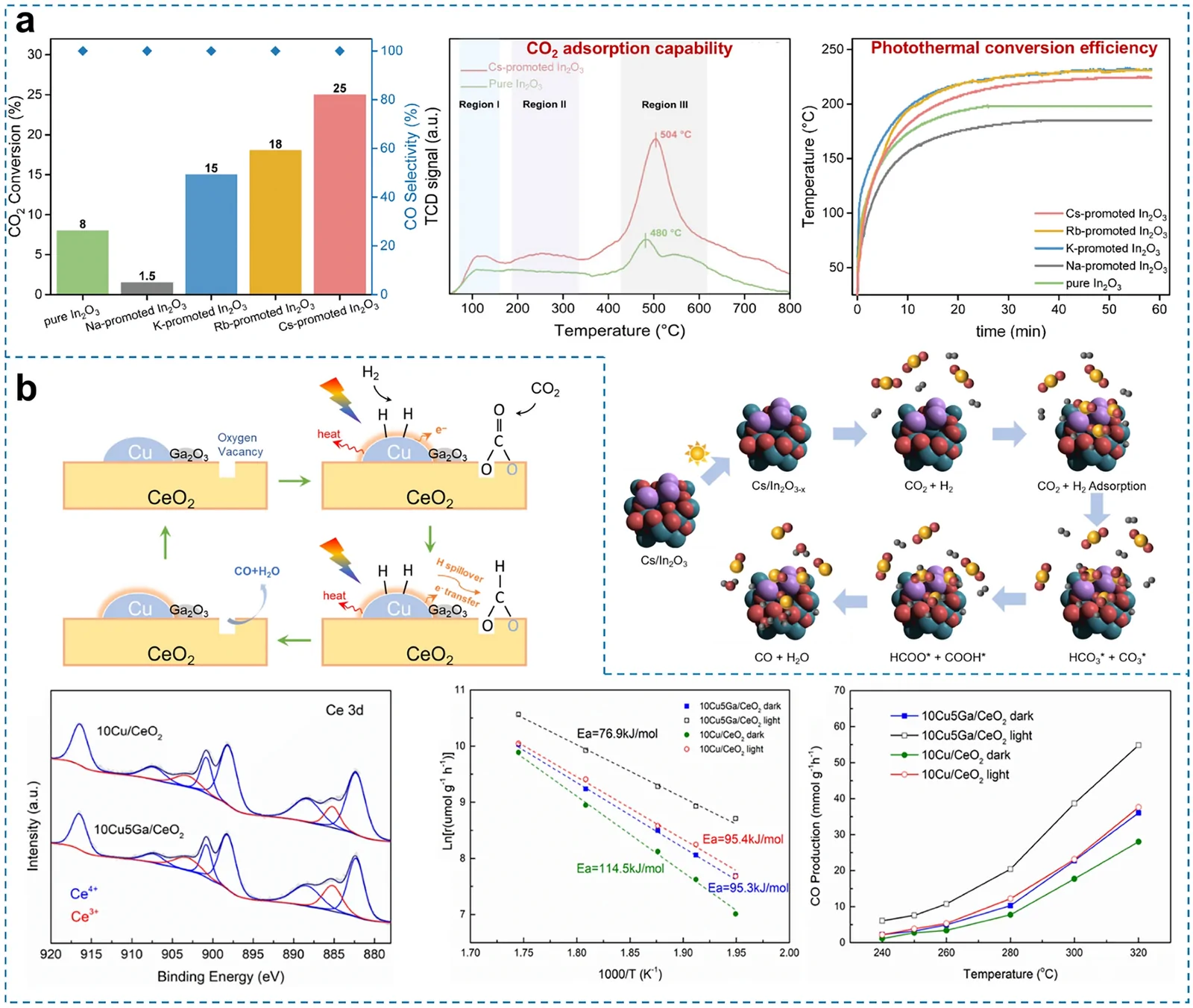
Effect of basic sites on facilitating the activation of CO_2_ to C_1_ products. **a** Alkali metal introduction in Cs-promoted In_2_O_3_. Reproduced with permission from Ref. [74], Copyright 2024 The Royal Society of Chemistry. **b** Transition metal oxides incorporation in 10Cu5Ga/CeO_2_. Reproduced with permission from Ref. [77], Copyright 2021 Elsevier

Alkali metal promoters can donate electron density to the catalyst surface and strengthen the electrostatic interaction with adsorbed CO_2_*, thereby facilitating CO_2_ adsorption and activation. Jorge Gascon et al. investigated a series of alkali metal-promoted In_2_O_3_ catalysts for selective photothermal CO_2_ hydrogenation to CO and found that Cs-promoted In_2_O_3_ exhibited the highest catalytic activity. This enhancement was attributed to strong electronic effects induced by Cs. Moreover, the surface temperatures of K-, Rb-, and Cs-promoted In_2_O_3_ catalysts were over 30 °C higher than that of pristine In_2_O_3_, indicating that alkali metal promoters also contribute to improved photothermal conversion efficiency (Fig. 11a) [74].

(2) Transition metal oxides

Certain transition metal oxides can serve as basic active sites by modulating surface charge density, thereby enhancing reactant activation. In Cu/CeO_2_ catalysts, the introduction of Ga_2_O_3_ altered the electronic structure and facilitated the reduction of CeO_2_, resulting in a higher concentration of oxygen vacancies that promote CO_2_ adsorption. In addition, Ga_2_O_3_ played a critical role in enabling hydrogen spillover, which accelerated the conversion of adsorbed carbonates into formates. These effects collectively contributed to the enhanced photothermal reverse water–gas shift (RWGS) performance (Fig. 11b) [77].

Basic sites enhance CO_2_ activation by strengthening catalyst–CO_2_ interactions and regulating surface electronic structure. However, excessive basicity may lead to strongly adsorbed carbonate species and slow intermediate conversion. Thus, efficient catalyst design requires a balance between CO_2_ adsorption strength, H_2_ activation ability, and photothermal energy utilization.

#### Creation of Surface Defects

Surface defect engineering strategies, particularly the introduction of oxygen vacancies and the creation of surface-frustrated Lewis pairs (SFLPs), hold significant promise for enhancing photothermal CO_2_ hydrogenation.

1. Oxygen vacancies

**Fig. 12:**
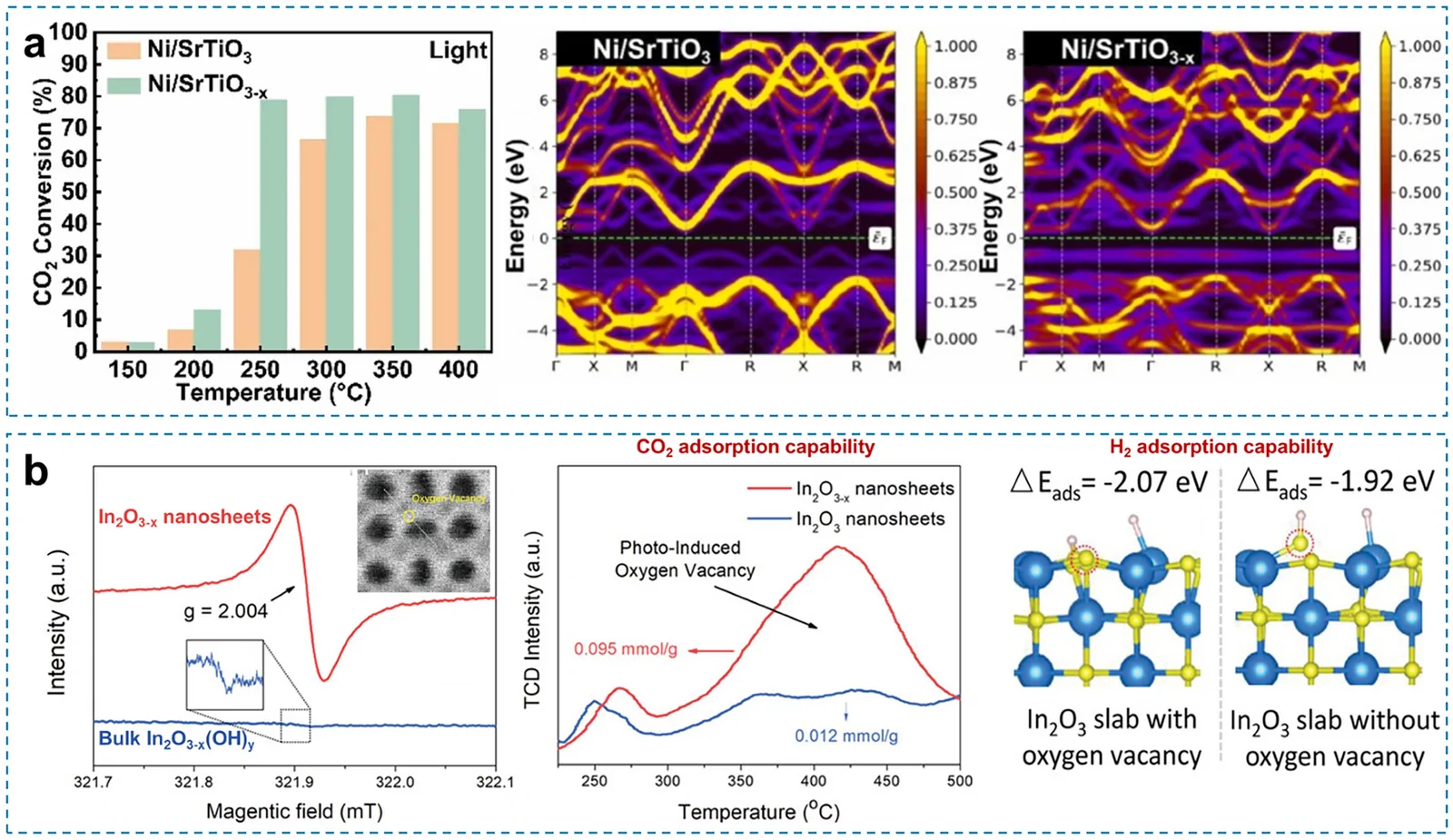
Effect of surface defects on facilitating the activation of CO_2_ to C_1_ products. **a** Oxygen vacancy creation in Ni/SrTiO_3−x_. Reproduced with permission from Ref. [162], Copyright 2024 Elsevier. **b** Oxygen vacancy creation in In_2_O_3−x_ nanosheets. Reproduced with permission from Ref. [71], Copyright 2019 John Wiley and Sons

The beneficial role of oxygen vacancies in promoting C_1_ product formation can be attributed to two main mechanisms. First, they enhance photothermal conversion by introducing mid-gap states and narrowing the band gap, thereby broadening solar absorption and facilitating photon utilization (Fig. 12a) [162]. Second, they substantially promote reactant activation, particularly for CO_2_ [108, 160]. Under reaction conditions, oxygen atoms from CO_2_ can fill oxygen vacancy sites by integrating into bridge-bonded oxygen, effectively healing the defect [163]. This process lowers the CO_2_ activation barrier due to strong attraction between oxygen vacancies and O atoms in CO_2_, thus facilitating CO_2_ activation. In addition, oxygen vacancies can trap photogenerated electrons, suppress charge recombination, and improve charge separation [162]. On black In_2_O_3−x_ nanosheets, oxygen vacancies generated localized electrons that were transferred to the antibonding orbitals of CO_2_, thereby stabilizing intermediates and facilitating CO_2_ activation. DFT calculations further revealed that oxygen vacancies reduced the dissociative adsorption energy of H_2_, promoting its activation and subsequent CO_2_ hydrogenation (Fig. 12b) [71].

However, oxygen vacancies may also introduce stability issues. Specifically, incorporation of oxygen atoms from CO_2_ into surface oxygen vacancies may hinder product desorption [164], and the continuous consumption of oxygen vacancies during reaction can lead to catalyst deactivation over time [165]. Therefore, future efforts should focus on strategies to enhance the durability and regeneration of oxygen vacancies in photothermal CO_2_ hydrogenation systems.

(2) Surface-frustrated Lewis pairs

Metal oxides usually contain surface Lewis acid–base pairs, but these sites often form stable Lewis adducts that are inactive toward CO_2_ activation. Introducing structural defects, especially oxygen vacancies, can disrupt local lattice order and expose adjacent Lewis acid–base sites. When the distance between these sites is appropriately regulated, typically around 4 Å, SFLPs can form and become active for small-molecule activation.

**Fig. 13:**
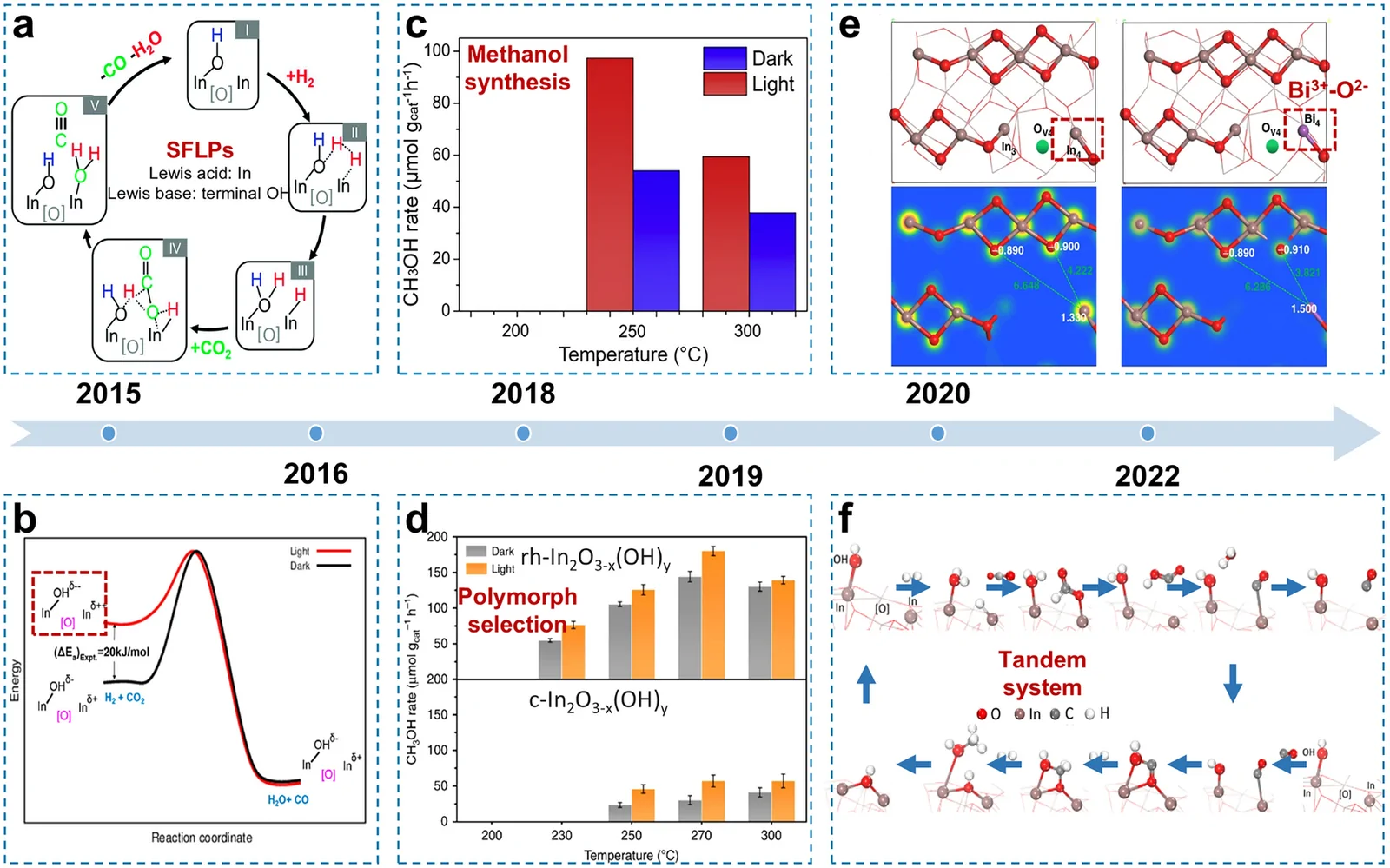
A brief overview of SFLP application in photothermal CO_2_ hydrogenation from the group of Geoffrey A. Ozin. **a** Proposed mechanism of SFLPs in In_2_O_3−x_(OH)_y_. Reproduced with permission from Ref. [70], Copyright 2015 The Royal Society of Chemistry. **b** Experimental and theoretical elucidation of SFLPs in In_2_O_3−x_(OH)_y_. Reproduced with permission from Ref. [167], Copyright 2016 American Chemical Society. **c** Promotion of photothermal CO_2_ to CH_3_OH by SFLPs In_2_O_3−x_(OH)_y_ nanocrystal superstructure. Reproduced with permission from Ref. [110], Copyright 2018 Elsevier. **d** Regulation of SFLPs acidity basicity via polymorph selection of In_2_O_3−x_(OH)_y_. Reproduced with permission from Ref. [113], Copyright 2019 Springer Nature. **e** Promotion of photothermal CO_2_ to CH_3_OH by SFLPs Bi^3+^−O^2−^ pairs in Bi_x_In_2−x_O_3_. Reproduced with permission from Ref. [114], Copyright 2020 Springer Nature. **f** Tandem photothermal CO_2_ hydrogenation to CH_3_OH enabled by SFLPs (InOH⋯In) in H_z_In_2_O_3−x_(OH)_y_. Reproduced with permission from Ref. [120], Copyright 2022 Springer Nature

In oxide-based SFLPs, the Lewis basic sites are often hydroxyl groups coordinated to metal atoms, while the Lewis acidic sites are coordinatively unsaturated metal cations (created by oxygen vacancies). Geoffrey A. Ozin et al. have conducted a series of studies investigating how SFLPs facilitate CO_2_ and H_2_ activation during photothermal CO_2_ hydrogenation. In In_2_O_3−x_(OH)_y_ (2015), adjacent hydroxyl groups and indium cations associated with oxygen vacancies formed molecular SFLPs that could heterolytically cleave H_2_ and reversibly adsorb CO_2_, enabling its conversion to CO (Fig. 13a) [70]. In 2016, In and InOH SFLPs were identified as active sites responsible for CO_2_ activation to CO (Fig. 13b) [166, 167]. In 2018, beyond CO production, the SFLP InOH⋯In in rod-shaped In_2_O_3−x_(OH)_y_ nanocrystal superstructures was found to facilitate CO_2_ hydrogenation to CH_3_OH under ambient pressure (Fig. 13c) [110]. In 2019, tuning the Lewis acidity and basicity of SFLPs through polymorph selection was found to regulate both activity and selectivity in photothermal CO_2_ hydrogenation (Fig. 13d) [113]. More recently, CH_3_OH production over H_z_In_2_O_3−x_(OH)_y_ was proposed to proceed through a tandem mechanism in which SFLPs, InOH⋯In sites, first heterolytically dissociated H_2_, followed by CO_2_ activation and conversion via the COOH* pathway. More importantly, the oxygen vacancy sites could be regenerated after CH_3_OH desorption, contributing to catalyst stability (Fig. 13f) [120]. Recently, Miao et al. further extended the SFLP concept beyond In_2_O_3_-based systems by showing that dynamic surface reconstruction of plasmonic Pt/TiN generated an oxygen vacancy-rich TiO_x_ shell, where surface oxygen vacancies and hydroxyl groups formed SFLPs that promoted CO_2_ adsorption and activation under photothermal conditions [168].

Besides hydroxyl-containing SFLPs, another SFLP type consists of coordinatively unsaturated metal cations as Lewis acidic sites and adjacent lattice oxygen atoms as Lewis basic sites [169]. In Bi_x_In_2−x_O_3_ (2020), isomorphic substitution of In^3+^ by Bi^3+^ generated Bi^3+^−O^2−^ SFLPs that strongly polarized H_2_ and promoted its dissociation into H* species, thereby promoting reactant activation (Fig. 13e) [114].

Overall, the activity of SFLPs originates from their cooperative dual-site character: Lewis acidic sites interact with the oxygen atoms of CO_2_, whereas Lewis basic sites facilitate polarization of the carbon-centered bonds or H_2_, thereby lowering the activation barrier for subsequent reactions. Although SFLPs are highly promising for defect engineering, achieving stable and durable SFLP configurations under photothermal reaction conditions remains a key challenge.

#### Introduction of Metal Active Sites

Introducing metal active sites can promote CO_2_ activation through strong orbital interactions with adsorbed molecules and facilitate H_2_ dissociation by modulating the local electronic structure, thereby enhancing reactant activation in photothermal CO_2_ hydrogenation. In some cases, metal active sites can also induce LSPR effects under light irradiation, generating localized heating and further improving photothermal catalytic performance.

1. Noble metal active sites

Among the various candidates, noble metals have been most extensively explored because of their outstanding ability to promote both charge transfer and reactant activation. Ir nanoparticles confined within the UiO-66 framework enabled efficient photothermal CO_2_ hydrogenation to CH_4_, achieving a production rate of 19.9 mmol g_cat_^−1^ h^−1^ by promoting charge separation and transfer, thereby accelerating H_2_ dissociation and CO_2_ activation [104].

**Fig. 14:**
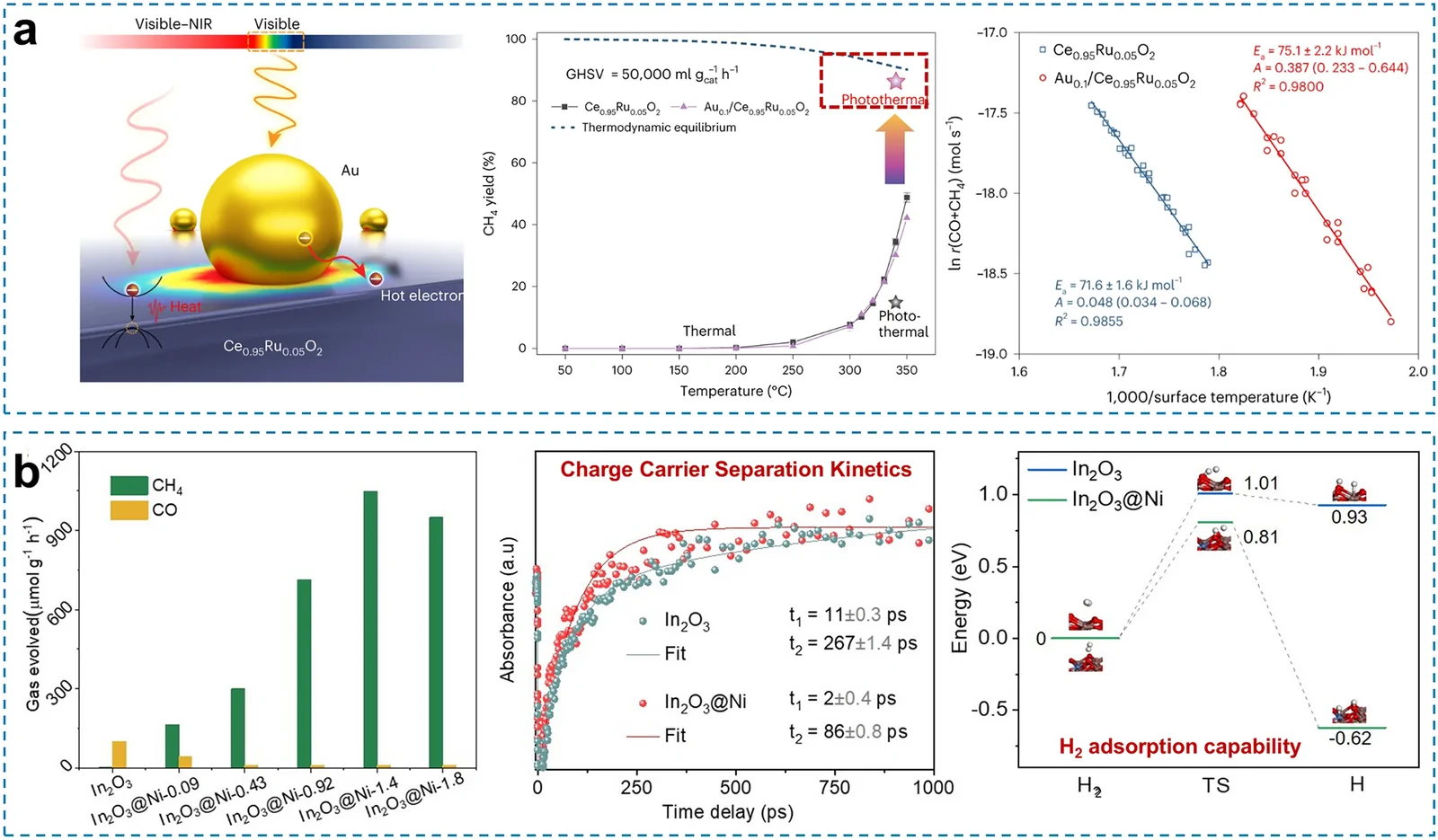
Effect of metal active sites on facilitating the activation of CO_2_ to C_1_ products. **a** Introduction of noble metal active sites Au in Ce_0.95_Ru_0.05_O_2_. Reproduced with permission from Ref. [44], Copyright 2023 Springer Nature. **b** Anchoring of transition metal active sites Ni onto In_2_O_3_. Reproduced with permission from Ref. [93], Copyright 2024 American Chemical Society

In addition to facilitating reactant activation, noble metal active sites can also enhance photothermal conversion through plasmonic effects. In an Au-grafted Ce_0.95_Ru_0.05_O_2_ solid solution catalyst, Ce_0.95_Ru_0.05_O_2_ acted as a broad-band light absorber to provide localized photothermal heating, while Au nanoparticles introduced visible-light LSPR at the Au/Ce_0.95_Ru_0.05_O_2_ interface. The role of Au was not simply assigned to additional heating, because Au_0.1_/Ce_0.95_Ru_0.05_O_2_ and Ce_0.95_Ru_0.05_O_2_ exhibited comparable surface temperatures under identical illumination. Arrhenius analysis showed similar apparent activation energies but an approximately 6–8-fold increase in the pre-exponential factor after Au loading, suggesting an increased number of effective active sites rather than pure Arrhenius thermal acceleration. Further analysis showed that LSPR-induced hot-electron injection promoted the formation of Ru–oxygen vacancy active centers, thereby enhancing CO_2_ methanation and enabling a CH_4_ yield of approximately 86%, close to the thermodynamic limit (Fig. 14a) [44]. A similar LSPR-assisted promotion was also reported for Ru/In_2_O_3_ in photothermal CO_2_ hydrogenation to methanol, where LSPR-induced hot carriers enhanced both surface heating and reactant activation [108].

(2) Transition metal active sites

Despite their high activity, the limited abundance and high cost of noble metals have driven increasing interest in transition metal alternatives. In this regard, transition metal active sites can also effectively regulate surface electronic structure and promote key elementary steps in photothermal CO_2_ hydrogenation. For instance, In_2_O_3_@Ni with uniformly dispersed Ni sites was reported to selectively catalyze CO_2_ hydrogenation to CH_4_, where the isolated Ni centers enhanced CO_2_ activation by modulating the surface electronic structure of In_2_O_3_, while also lowering the barrier for H_2_ dissociation to provide reactive hydrogen species (Fig. 14b) [93].

Furthermore, when Ni species present as metallic nanoparticles, they not only facilitated reactant mass transfer but also served as local thermal hotspots via the LSPR effect, providing an additional contribution to photothermal catalytic performance [170, 171].

Metal active sites enhance photothermal CO_2_ hydrogenation by improving H_2_ dissociation, CO_2_ activation, interfacial charge transfer, and, in some systems, plasmon-assisted light utilization. Noble metals usually show higher intrinsic activity, whereas transition metals provide lower-cost alternatives with tunable electronic structures. Nevertheless, metal aggregation, uncertain active-state evolution, and the difficulty in distinguishing thermal and electronic contributions remain important challenges. Therefore, future studies should combine metal-site stabilization with operando characterization and temperature-matched controls.

### Promoting C−C Coupling of C_1_ Intermediates to C_2+_ Products

The coupling of C_1_ intermediates to form C−C bonds is a key step governing both the rate and selectivity of C_2+_ products in photothermal CO_2_ hydrogenation. Accordingly, catalyst design has increasingly focused on strengthening C−C coupling along distinct C_1_-mediated pathways to produce multicarbon products.

#### CO* Coupling

Achieving efficient C_2_ product formation through the CO*-mediated pathway requires not only sufficient CO* coverage, but also favorable adsorption configurations and electronic environments for subsequent C−C coupling. Accordingly, current catalyst design strategies have mainly focused on alloy active sites, charge-polarized dual-active centers, and bifunctional catalyst systems.

1. Fabrication of alloy active sites

**Fig. 15:**
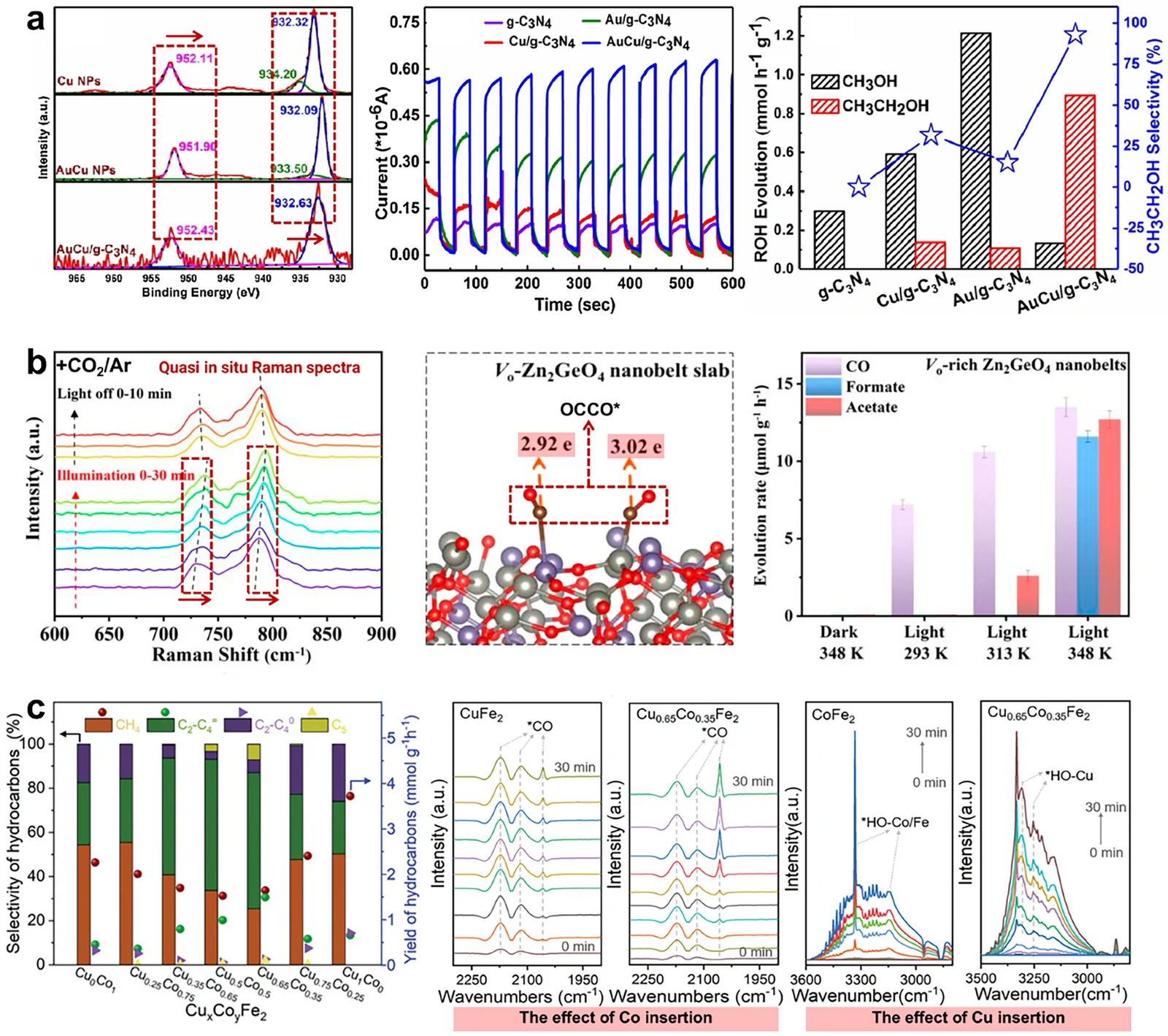
Surface optimization strategies for promoting C−C coupling of C_1_ intermediates via CO* coupling. **a** Fabrication of AuCu alloy sites to facilitate CO*−CO* coupling. Reproduced with permission from Ref. [136], Copyright 2020 Elsevier. **b** Design of charge-polarized Zn−O−Ge to promote CO*−CO* coupling. Reproduced with permission from Ref. [137], Copyright 2021 American Chemical Society. **c** Construction of bifunctional catalyst Cu/CoFe to promote CO*−HCO* coupling. Reproduced with permission from Ref. [135], Copyright 2024 John Wiley and Sons

Alloying is an effective strategy for promoting C_2+_ formation as the introduction of a secondary metal can tune the electronic and geometric structure of the host metal, thereby strengthening CO_2_ adsorption and facilitating its conversion to CO* intermediates [172]. The resulting increase in surface CO* coverage is beneficial for subsequent C−C coupling. In AuCu alloy nanoparticles supported on g-C_3_N_4_ nanosheets, the alloy interface played a central role in regulating the surface electronic structure, leading to electron enrichment at Cu sites and therefore more efficient activation of CO_2_ to CO. Coupled with accelerated carrier migration at the alloy–support interface and the higher collision frequency of CO* intermediates under photothermal heating, this alloying effect promoted CO*−CO* coupling and enhanced ethanol production (Fig. 15a) [136].

(2) Design of charge-polarized dual-active sites

Charge-polarized dual-active sites can facilitate C−C bond formation by creating asymmetric electronic environments that reduce the electrostatic repulsion between neighboring C_1_ intermediates [173–176]. In an In−S_V_−Bi active site, the rational spatial arrangement and asymmetric electronic environment between In and Bi atoms facilitated the coupling of CO* intermediates into OCCO*, lowering energy barriers for both CO_2_ activation and C−C coupling [177]. In oxygen vacancy-rich Zn_2_GeO_4_ nanobelts, quasi-in situ Raman spectroscopy directly identified Zn−O−Ge as active site for photothermal catalysis, enabling simultaneous CO_2_ adsorption on both Zn and Ge atoms. The presence of oxygen vacancies further amplified the electronic asymmetry of these Zn−O−Ge sites, resulting in uneven charge distribution across neighboring CO* species. Combined with the higher collision probability of CO* intermediates under photothermal heating, this vacancy-enhanced asymmetry facilitated C−C coupling and favored acetate formation, affording an acetate selectivity of 66.9% and a CO_2_ conversion ratio of 29.95% (Fig. 15b) [137].

(3) Construction of bifunctional catalysts

The synergy between two complementary functions is particularly beneficial for C_2+_ olefin formation, especially when one component promotes the generation and coupling of carbon-containing intermediates, while the other suppresses excessive hydrogenation. This cooperative effect was realized in a Cu-promoted CoFe alloy catalyst, which delivered a C_2+_ hydrocarbon selectivity of 73.7%, including 56.5% selectivity toward C_2_−C_4_ olefins. In this system, Cu enhanced CO_2_ adsorption and facilitated hydrogen spillover, which modulated the surface hydrogenation behavior and inhibited further hydrogenation of carbon-containing intermediates. Meanwhile, Co in the CoFe alloy favored bridged CO* adsorption and, together with Fe, provided stable sites for C_1_ intermediate coupling, thereby promoting C−C bond formation and favoring C_2_−C_4_ olefin production (Fig. 15c) [135].

In summary, CO*-mediated coupling is highly dependent on sufficient CO* coverage and suitable adsorption configurations. Alloying, charge-polarized dual-active sites, and bifunctional catalyst design can enhance this process by regulating CO_2_ activation, CO* adsorption, interfacial charge distribution, and hydrogenation behavior. Nevertheless, the key challenge is to avoid either excessive CO* stabilization or rapid hydrogenation to C_1_ products. Thus, effective CO* coupling catalysts should simultaneously promote CO* formation, maintain appropriate intermediate mobility, and provide favorable sites for C–C bond formation.

#### ***CH***_x_* Coupling

As one of the most extensively investigated routes toward C_2+_ products in photothermal CO_2_ hydrogenation, the CH_x_*-mediated pathway places demands on both the surface availability of CH_x_* intermediates and the catalytic environment required for their subsequent coupling. In response, catalyst design has been directed mainly toward metal–oxide interfaces, alloy active sites, and charge-polarized dual-active sites.

1. Formation of metal**–**oxide interfaces

**Fig. 16:**
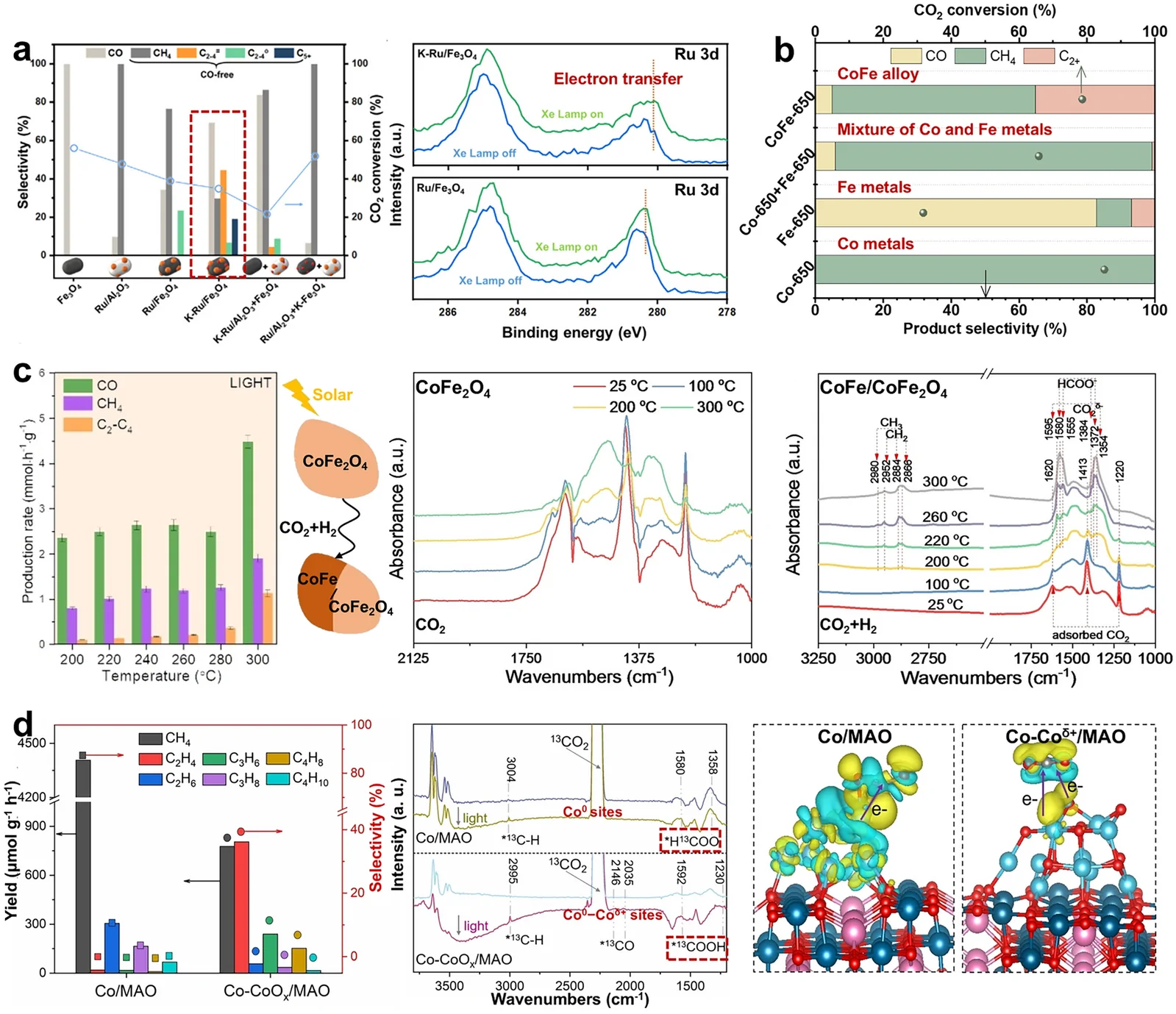
Surface optimization strategies for promoting C−C coupling of C_1_ intermediates via CH_x_* coupling. **a** Formation of Ru−FeO_x_ interface to facilitate CH_2_*−CH_2_* coupling. Reproduced with permission from Ref. [127], Copyright 2024 Elsevier. **b** Fabrication of CoFe alloy active sites to facilitate CH*−CH_2_* coupling. Reproduced with permission from Ref. [128], Copyright 2017 John Wiley and Sons. **c** Fabrication of CoFe alloy active sites to facilitate C−C coupling. Reproduced with permission from Ref. [133], Copyright 2023 John Wiley and Sons. **d** Design of charge-polarized Co^0^−Co^δ+^ to facilitate CH*−CH* coupling. Reproduced with permission from Ref. [134], Copyright 2023 John Wiley and Sons

The formation of metal–oxide interfaces can reshape interfacial charge transfer and thereby favor C−C coupling in photothermal CO_2_ hydrogenation. In a K−Ru/Fe_3_O_4_ catalyst, while CO_2_ activation and hydrogenation predominantly occurred on Ru sites, Ru−FeO_x_ interface served as the key site for C_2+_ product generation. Upon light irradiation, photoexcited electrons in Fe_3_O_4_ were transferred across the Ru−FeO_x_ interface to Ru, increasing electron accumulation at the interface and promoting C−C coupling (Fig. 16a). Meanwhile, K not only facilitated the construction of the Ru−FeO_x_ interface but also increased its electron density, which lowered the charge state of Ru, ultimately improving C_2_H_4_ selectivity [127].

(2) Fabrication of alloy active sites

Tuning the interaction between the catalyst surface and CH_x_* intermediates is crucial for promoting C−C coupling toward C_2+_ hydrocarbons. In this regard, CoFe alloy sites are particularly effective because alloying modifies the electronic structure relative to monometallic Co or Fe, optimizes the adsorption strength of CH_x_* species, and thus favors the balance between intermediate stabilization and further hydrogenation [178]. In a CoFe catalyst derived from CoFeAl-layered double-hydroxide nanosheets, this electronic modulation lowered the barrier for the CH*−CH_2_* coupling step compared with individual Co, Fe, or their physical mixtures, leading to more efficient C−C bond formation (Fig. 16b) [128]. Within a CoFe/CoFe_2_O_4_ heterostructure, CoFe alloy sites also played an important role in promoting C_2_−C_4_ hydrocarbon formation, giving a production rate of 1.1 mmol g^−1^ h^−1^ with 29.8% selectivity at a CO_2_ conversion of 12.9% under ambient pressure. In this system, CO_2_ activation occurred on CoFe_2_O_4_ to form adsorbed CO_2_* species, while the CoFe alloy promoted hydrocarbon chain growth via C−C coupling (Fig. 16c) [133].

(3) Design of charge-polarized dual-active sites

Charge-polarized dual-active sites can effectively promote CH_x_*-mediated C−C coupling by maintaining a sufficiently high surface population of CH_x_* intermediates while suppressing their excessive hydrogenation. This balance was effectively achieved on MgAl_2_O_4_-supported Co^0^−Co^δ+^ dual sites, where light irradiation enabled a C_2_−C_4_ hydrocarbon production rate of 1303 μmol g^−1^ h^−1^ with a carbon selectivity of 62.5%. The electronic contrast between Co^0^ and Co^δ+^ sites shifted the reaction pathway from HCOO* to COOH*, thereby favoring olefin formation. In this system, Co^0^ promoted CO_2_ adsorption and activation to generate C_1_ intermediates, whereas electron-deficient Co^δ+^ lowered the barrier for CHCH* formation and, by withdrawing electron density from neighboring Co^0^ sites, inhibited the over-hydrogenation of CH_x_* intermediates (Fig. 16d) [134]. The same design logic was also effective in Fe/FeO_x_/Al_2_O_3_−MgO, which achieved a C_2+_ hydrocarbon selectivity of 52.9% under ultraviolet–visible illumination. There, FeO_x_ suppressed further hydrogenation of CH_2_* and CH_3_* intermediates on Fe^0^ sites, thus increasing the surface concentration of CH_x_* species and facilitating C−C coupling [129].

Overall, charge-polarized dual-active sites create asymmetric charge environments for C_1_ intermediates through their distinct valence states and electron densities, thereby promoting C−C coupling while suppressing excessive hydrogenation. In particular, electron-deficient metal species can act as electron acceptors to inhibit further hydrogenation of C_1_ intermediates and thus favor C−C bond formation [169].

#### ***CH***_***3***_OH* Coupling

As direct studies on CH_3_OH*-mediated C−C coupling in photothermal CO_2_ hydrogenation remain unavailable, insights from thermal catalysis provide a useful basis for catalyst design. Current efforts have centered on tandem systems that integrate metal oxides with zeolites, where the former are responsible for generating oxygenated intermediates and the latter provide confined acidic environments for subsequent C−C bond formation. Within such systems, the efficiency of multicarbon product formation is governed largely by pore architecture and acidity of the zeolite, as well as spatial intimacy between the two catalytic components.

1. Regulation of pore size structure

**Fig. 17:**
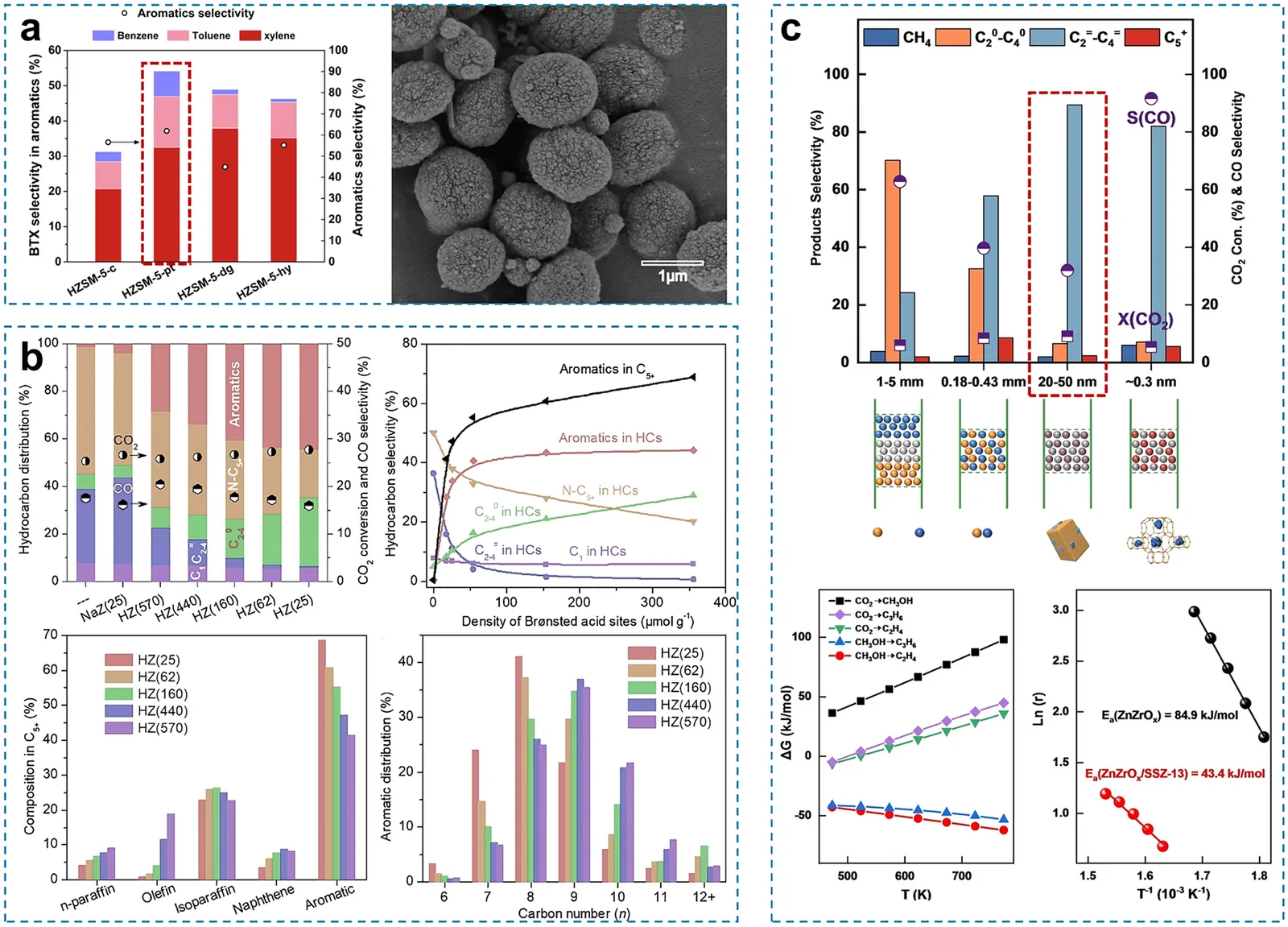
Surface optimization strategies for promoting C−C coupling of C_1_ intermediates via CH_3_OH* coupling. **a** Pore size structure regulation in HZSM-5. Reproduced with permission from Ref. [181], Copyright 2020 American Chemical Society. **b** Zeolite acidity adjustment in HZ. Reproduced with permission from Ref. [184], Copyright 2021 Elsevier. **c** Proximity effect optimization in ZnZrO_x_/SSZ-13. Reproduced with permission from Ref. [143], Copyright 2024 John Wiley and Sons

Tuning the pore architecture of zeolite catalysts significantly influences the C−C coupling process, primarily through confinement effects. Properly sized microporous environments can spatially restrict the diffusion of CO_2_, H_2_, and key hydrogenated intermediates (e.g., CO*, HCO*, CH_x_*), thereby enriching their local concentration and enhancing the probability of C−C bond formation [179]. In this regard, zeolites with 10-membered rings provide a more suitable confinement environment for the oligomerization of hydrocarbon pool species, whereas 8- or 12-membered-ring frameworks tend to impose either overly strong or insufficient confinement, making them less favorable for C−C bond formation [180]. Structural variations introduced during zeolite synthesis can further affect product selectivity. In a Cu-modified Fe_2_O_3_/HZSM-5 system, HZSM-5 prepared by a phase transfer method showed improved performance for the selective formation of benzene, toluene, and xylene, which was associated with the abundant mesopores (5−12 nm) that facilitated molecular diffusion and intermediate stabilization (Fig. 17a) [181].

(2) Adjustment of zeolite acidity

Brønsted acid sites within zeolites play a critical role in promoting the formation of C_2+_ products, as they catalyze essential carbon chain growth reactions, including alcohol dehydration, olefin oligomerization/polymerization, and alkylation with aromatics. This acidity can be finely regulated through the Si/Al ratio, which determines both the density and strength of Brønsted acid sites [182, 183]. Higher acid-site density generally favors the formation of aromatics and heavier olefins, whereas weaker or less abundant acid sites are more conducive to lighter olefins (Fig. 17b). Such differences are attributed to the higher catalytic activity of strongly acidic zeolites in facilitating olefin-to-aromatic conversions, which simultaneously generated reactive hydrogen species via olefin dehydro-aromatization. These hydrogen species can be consumed in CO_2_ or CO reduction, accelerating the RWGS and Fischer–Tropsch synthesis processes. However, overly strong acidity may lead to excessive cracking, thereby reducing the selectivity toward C_5+_ products [184].

(3) Optimization of proximity effect

The efficiency of C_2+_ hydrocarbon formation in oxide–zeolite tandem systems is governed to a large extent by how rapidly oxygenated intermediates, such as CH_3_OH*, can migrate from metal–oxide sites to Brønsted acid sites in the zeolite. Therefore, the proximity effect between the two components is a decisive factor influencing both activity and selectivity [185].

In the ZnZrO_x_/SSZ-13 system, a separation distance of 20−50 nanometers was found to be most favorable for light-olefin production (Fig. 17c). Although shorter distances may increase local intermediate concentrations, they also risk pore blockage, reduced surface area, and restricted diffusion, ultimately impairing hydrocarbon production. By contrast, excessively large separations hinder intermediate transfer and lower overall conversion efficiency. A well-balanced proximity can enhance proton mobility and intermediate diffusion, increasing local concentration of carbon species and facilitating subsequent chain growth. Moreover, compared to stepwise operation, this tandem route via CH_3_OH* intermediate is both thermodynamically and kinetically advantageous [143].

Compared with CO* or CH_x_* coupling routes, systems involving CH_3_OH* intermediates require more complex catalyst design, yet they offer a distinct advantage in breaking away from the conventional ASF distribution, allowing for higher selectivity toward C_2+_ products. Although CH_3_OH*-mediated C−C coupling has not yet been demonstrated in photothermal CO_2_ hydrogenation, the incorporation of zeolites into photothermal catalytic systems has already begun to attract attention [186]. Hence, integrating oxide–zeolite tandem architectures into photothermal CO_2_ hydrogenation may provide a promising route toward higher activity and improved C_2+_ selectivity.

Overall, as compared in Table 6, these strategies indicate that efficient C_2+_ synthesis requires the cooperative regulation of upstream CO_2_ activation and downstream C–C coupling. Future catalyst design should therefore integrate photothermal field regulation, active-site construction, intermediate stabilization, and coupling-site engineering rather than optimizing a single material feature alone.

**Table 6:** Comparison of catalyst modification strategies for photothermal CO_2_ hydrogenation toward C_2+_ products

Strategy	Advantages	Challenges	Application scope
Control of morphology	Most useful when light absorption or heat delivery limits the rate Provides an engineering basis for flow and outdoor reactors	Rate gains may be purely thermal without matched temperature controls Hot spots can induce sintering, coking, and spatial selectivity gradients	RWGS/CO generation, methanation, and C_1_ precursor formation Requires additional C−C coupling sites for selective C_2+_ synthesis
Construction of basic sites	Builds reactive C_1_ precursor pools using simple promoter chemistry Compatible with many metal and oxide catalysts	Strong basicity can trap carbonate/formate species Provides little intrinsic C−C coupling ability	CO and methanol precursor formation Auxiliary module for C_2+_ catalysts when paired with coupling sites
Creation of surface defects	Links light absorption, carrier trapping, and oxide surface chemistry Can tune COOH*/HCOO* pathway preference	Defects and SFLPs are dynamic under CO_2_/H_2_/light/heat Assignments often need operando, isotope, or regeneration evidence	Oxide-mediated CO and methanol routes C_2+_ use requires stable, regenerable defects
Introduction of metal active sites	Strong gas activation at low temperature or under light Useful models for separating thermal, plasmonic, and carrier effects	High hydrogenation activity can favor CH_4_ over C_2+_ products Cost, sintering, and hot-carrier ambiguity limit generality	Methanation, methanol formation, and upstream C_1_ activation Needs coupling control sites for C_2+_ selectivity
Alloy-mediated coupling-site engineering	Directly links composition to C−C coupling probability Can balance CO_2_ activation, H supply, and chain growth	Active ensembles are phase- and composition-sensitive Segregation, oxidation, or excess H coverage can redirect selectivity	CO*- or CH_x_*-mediated ethanol, acetate, and C_2_−C_4_ hydrocarbons Requires operando identification of the active alloy/interface state
Interface and bifunctional engineering	Balances C_1_ generation with suppression of overhydrogenation Particularly valuable for olefin-oriented systems	Performance is highly proximity-dependent Interface reconstruction and multiple active phases complicate mechanism assignment	CO*−HCO*/CO*−COH* coupling and CH_x_*-mediated olefin routes Systems needing balanced activation, spillover, and hydrogenation control
Charge-polarized dual-site engineering	Targets the intrinsic C−C coupling bottleneck directly Can bias selectivity toward light olefins, ethanol, or acetate	Precise, stable paired-site construction remains difficult Similar trends may also arise from alloys, interfaces, or thermal coverage effects	High-selectivity C_2_ design via CO*−CO* or CH_x_*−CH_x_* coupling Best used with operando spectroscopy and DFT verification
Tandem and confinement engineering	Bypasses ASF-type limitations Offers a clear route to olefin and aromatic selectivity	Direct photothermal C_2+_ evidence remains limited Coking, diffusion limits, and heat transfer mismatch can erode performance	CH_3_OH*/oxygenate-mediated olefins, aromatics, and liquid fuels Hybrid thermal photothermal tandem systems

## Scale Demonstration of Photothermal CO_2_ Hydrogenation

The long-term appeal of photothermal CO_2_ hydrogenation to C_2+_ products lies in its potential to enable efficient and value-added CO_2_ conversion using solar energy as the sole energy input, while avoiding secondary pollution. Recent studies have increasingly moved beyond laboratory-scale evaluation toward demonstrations under direct sunlight, aiming to assess the scalability of photothermal CO_2_ hydrogenation. However, most scale demonstrations reported so far remain centered on CO, CH_4_, or methanol. These C_1_-oriented scale systems are still important as they provide key intermediates and engineering experience for downstream C–C coupling. Thus, this section highlights representative scale demonstrations as useful references for the future scale-up of photothermal CO_2_-to-C_2+_ conversion.

### Scale Demonstrations of Catalytic CO_2_ Hydrogenation Enabled by Optical Concentration-Enhanced Solar Energy Harvesting

When solar energy serves as the sole energy input, the local temperature generated on photothermal catalysts under natural sunlight is often insufficient to reach the optimal range required for efficient CO_2_ hydrogenation, even for materials with high photothermal conversion efficiency. To overcome this limitation, solar concentrators have been introduced to intensify incident sunlight, thereby elevating catalyst surface temperatures to levels suitable for efficient reaction performance [187, 188].

**Fig. 18:**
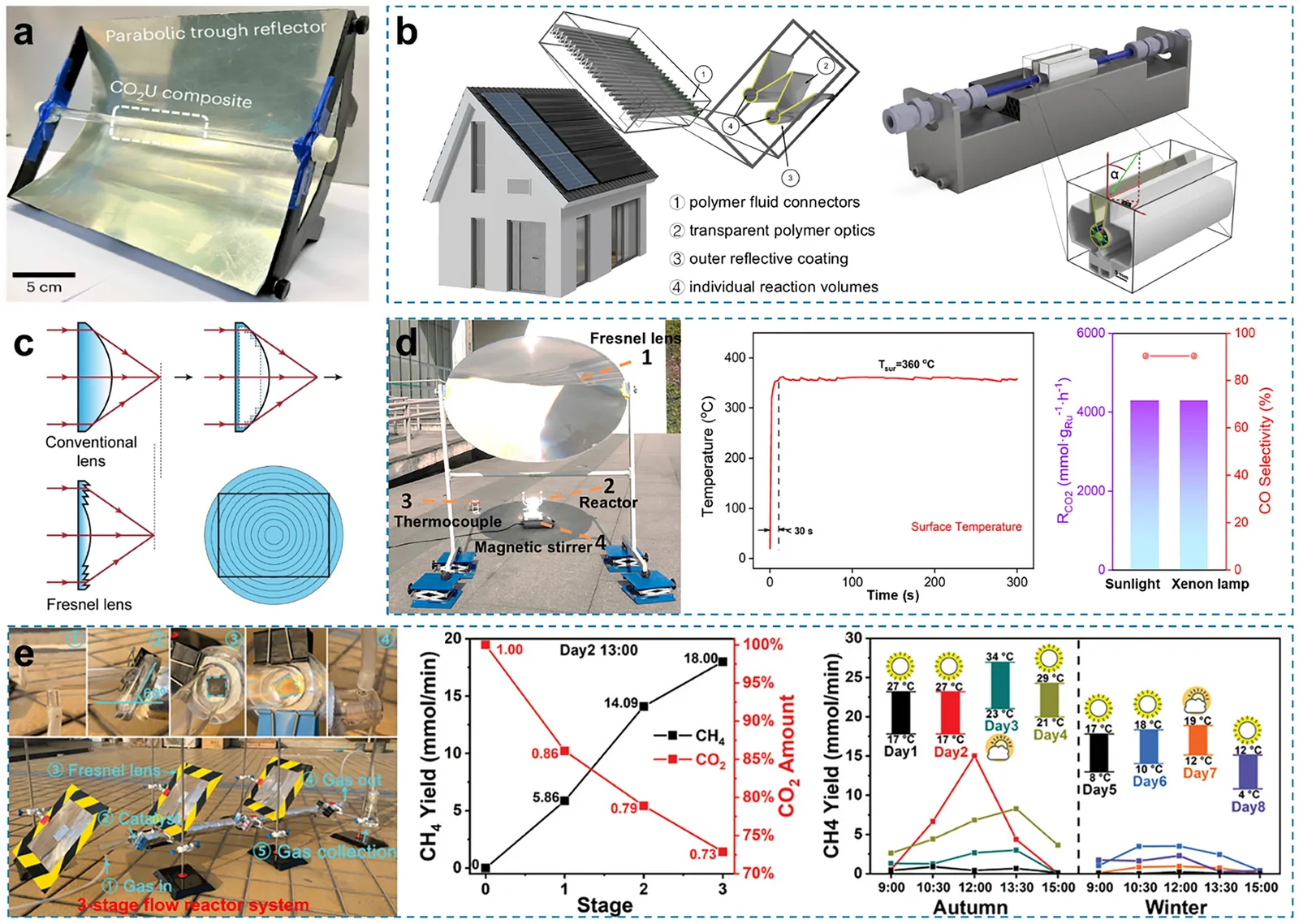
Scale demonstrations of photothermal CO_2_ hydrogenation enabled by optical concentration-enhanced solar energy harvesting. **a** Parabolic trough concentrator. Reproduced with permission from Ref. [190], Copyright 2025 Springer Nature. **b** V-shaped solar concentrator for solar-driven synthesis. Reproduced with permission from Ref. [191], Copyright 2023 Elsevier. **c** Comparison between conventional lens and Fresnel lens. Reproduced with permission from Ref. [81], Copyright 2023 John Wiley and Sons. **d** Fresnel lens-based concentrator on Ru/Mo_2_TiC_2_. Reproduced with permission from Ref. [193], Copyright 2022 American Chemical Society. **e** Three-stage flow Fresnel lens-based concentrator on Ru-Al_2_O_3−x_-L. Reproduced with permission from Ref. [194], Copyright 2022 John Wiley and Sons

Among the available designs, parabolic trough concentrators enhance local irradiance by focusing sunlight onto a linear focal region, thereby improving photothermal reaction efficiency [189]. Under such concentrated irradiation, light intensity can reach 300 mW cm^−2^, giving a surface temperature of about 56 °C on γ-Al_2_O_3_|TiO_2_|CotpyP (Fig. 18a). To prevent catalyst sintering and coking under intensified heating, water-jacketed cooling systems can be incorporated into reactor designs to maintain operating conditions below deactivation thresholds [190]. Additionally, panel-type photoreactors integrating V-shaped concentrators with tubular reaction cavities have also been developed as alternative solar concentration configurations. This configuration, composed of hundreds of parallel reaction channels, enabled a more uniform photon flux distribution within the active zone, improved quantum efficiency, and showed promise for decentralized rooftop-scale applications (Fig. 18b) [191].

Unlike traditional convex lenses, Fresnel lenses retain the refractive geometry while removing internal bulk material, thereby reducing thickness and shortening focal length (Fig. 18c) [192]. The reduced thickness minimizes internal light absorption, allowing for cost-effective production using materials such as plastic [81].

Applied to a Ru/Mo_2_TiC_2_ catalyst under outdoor conditions, the Fresnel lens increased the incident intensity to 3.7 W cm^−2^ and raised catalyst temperature to ~360 °C within 30 s, resulting in an outdoor CO production rate of 3.9 mol g_Ru_^−1^ h^−1^ with 90% selectivity (Fig. 18d) [193]. Building on this concept, a three-stage flow reactor was further developed to improve overall CO_2_ conversion under outdoor operation. As the reactant stream passed sequentially through three catalytic zones containing Ru-Al_2_O_3−x_-L microchannel catalysts, the product yield increased progressively, while the CO_2_ concentration decreased, ultimately giving a CO_2_ conversion of 27.10% and a CH_4_ production rate of 18.00 mmol min^−1^ (Fig. 18e). However, performance remains closely tied to solar availability: the highest product yields were observed at around 13:30 in autumn and 12:00 in winter [194], and product formation rates under clear skies were nearly twice those measured under cloudy conditions [195].

### Scale Demonstrations of Catalytic CO_2_ Hydrogenation Enabled by Solar-to-Thermal Energy Conversion

An alternative route for scaling up solar-driven photothermal CO_2_ hydrogenation relies on solar-to-thermal conversion devices that harvest sunlight, store it as heat, and deliver the accumulated thermal energy to the catalytic reactor. This solar-to-thermal conversion pathway offers a practical means to stabilize reaction conditions while exploiting renewable solar input.

**Fig. 19:**
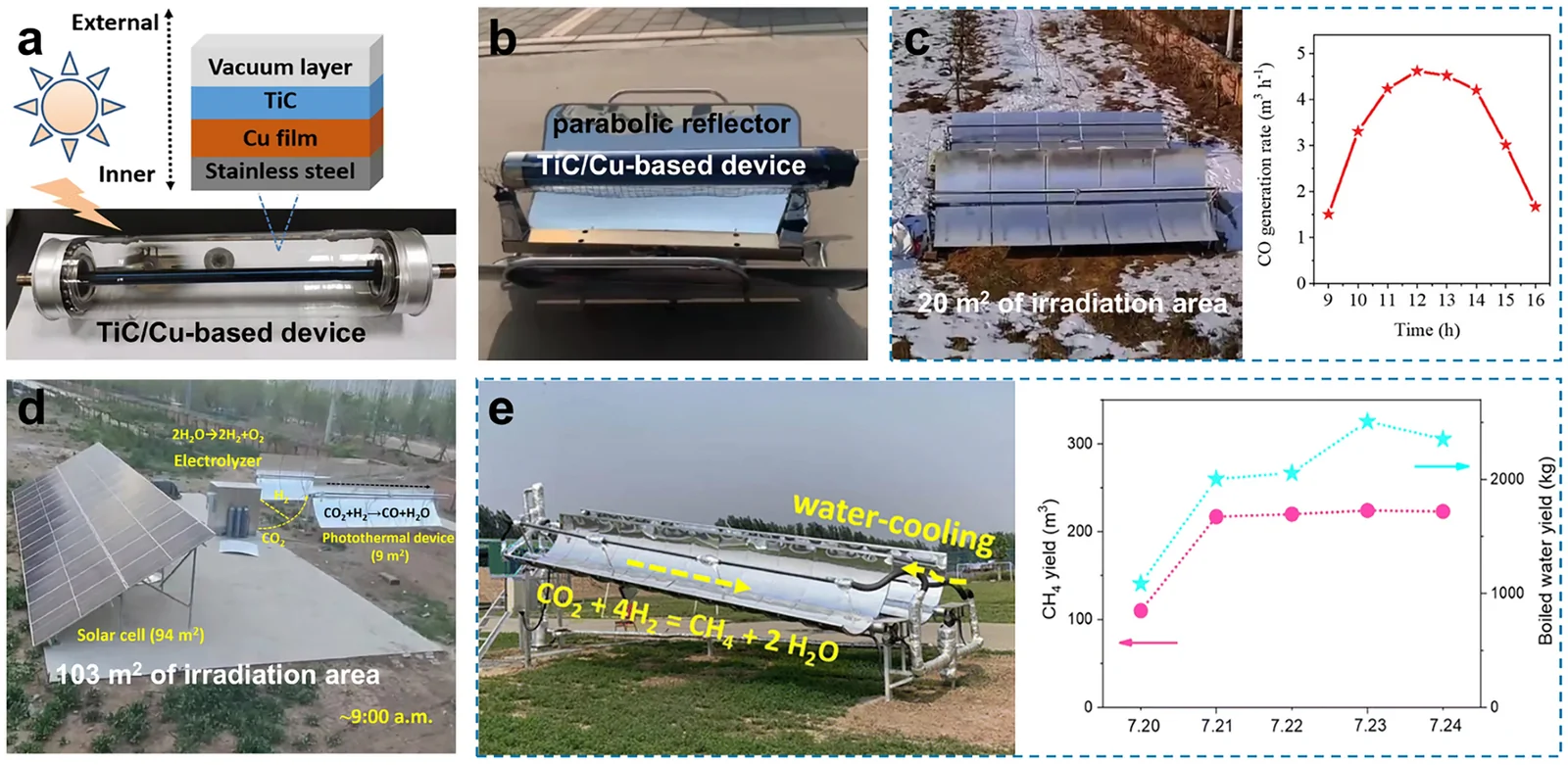
Scale demonstrations of photothermal CO_2_ hydrogenation enabled by solar-to-thermal energy conversion. **a** TiC/Cu-based solar-to-thermal devices. Reproduced with permission from Ref. [198], Copyright 2025 The Royal Society of Chemistry. **b** TiC/Cu-based solar-to-thermal devices on Cu_2_Zn_1_Al_0.5_Ce_5_Zr_0.5_O_x_. Reproduced with permission from Ref. [68], Copyright 2023 Springer Nature. **c** 20 m^2^ TiC/Cu-based solar-to-thermal devices on AgK/Fe_3_O_4_. Reproduced with permission from Ref. [64], Copyright 2024 John Wiley and Sons. **d** 103 m^2^ TiC/Cu-based solar-to-thermal devices on Ni_1_Ag_0.02_O_1_. Reproduced with permission from Ref. [197], Copyright 2024 The Authors. **e** Self-sustained TiC/Cu-based solar-to-thermal devices on Ni_1_Ce_1_O_3_. Reproduced with permission from Ref. [198], Copyright 2025 The Royal Society of Chemistry

Since 2022, a series of scale demonstrations based on solar-to-thermal energy conversion have been reported. Early work employed a Bi_2_Te_3_/Cu-based device for photothermal methanol reforming to hydrogen, in which Bi_2_Te_3_ served as an efficient solar absorber capable of converting up to 99% of incident light into heat, while the highly conductive Cu layer suppressed infrared radiative loss. As a result, the device reached an internal temperature of 307 °C under one-sun illumination [196]. In 2023, TiC/Cu-based devices were employed in outdoor demonstrations of photothermal CO_2_ hydrogenation, with TiC assumed to function analogously to Bi_2_Te_3_ (Fig. 19a). Under one-sun illumination, the enclosed catalyst could reach surface temperatures of 350–380 °C, providing sufficient thermal energy to drive the CO_2_ hydrogenation reaction solely via solar-to-thermal conversion.

This TiC/Cu platform was subsequently extended to progressively larger outdoor demonstrations. In a small-scale system using Cu_2_Zn_1_Al_0.5_Ce_5_Zr_0.5_O_x_ catalyst, a parabolic reflector was incorporated to concentrate diffuse sunlight and maintain elevated operating temperatures throughout the day (Fig. 19b) [68]. The same strategy was then applied to a 20 m^2^ illuminated area with AgK/Fe_3_O_4_ as the catalyst, where substantial CO production was sustained even at ambient temperatures below 10 °C, demonstrating reliable operation under relatively harsh outdoor conditions (Fig. 19c) [64]. Further scale-up to a 103 m^2^ field was achieved in 2024, together with photovoltaic water electrolysis as the hydrogen source, thereby establishing an artificial photosynthetic system for outdoor syngas production. This integrated system produced about 21.14 m^3^ of green syngas with an H_2_/CO ratio close to 1, demonstrating promising efficiency, scalability, and economic potential for outdoor application (Fig. 19d) [197]. More recently, this solar-to-thermal strategy was extended to a self-sustained continuous demonstration of photothermal CO_2_ methanation. Owing to the exothermic nature of CO_2_ methanation, the reaction could continue even in the dark once sufficient heat had been accumulated, and a water-cooling system was introduced to maintain the catalyst surface at 300 °C and avoid thermal deactivation. This design enabled continuous day-and-night operation while simultaneously generating usable hot water, further broadening the practical prospects of solar-driven photothermal CO_2_ hydrogenation (Fig. 19e) [198].

### Scale Demonstration of Catalytic CO_2_ Hydrogenation Enabled by Solar-to-Chemical Energy Conversion

Scale demonstrations of thermal catalytic CO_2_ hydrogenation based on solar-to-chemical energy conversion represent an emerging paradigm for sustainable fuel synthesis. In this approach, solar energy is initially stored in molecular carriers such as H_2_, which subsequently serve as energy carriers to drive catalytic CO_2_ conversion. This pathway not only facilitates continuous operation under fluctuating solar input but also addresses the challenge of securing a green hydrogen supply.

**Fig. 20:**
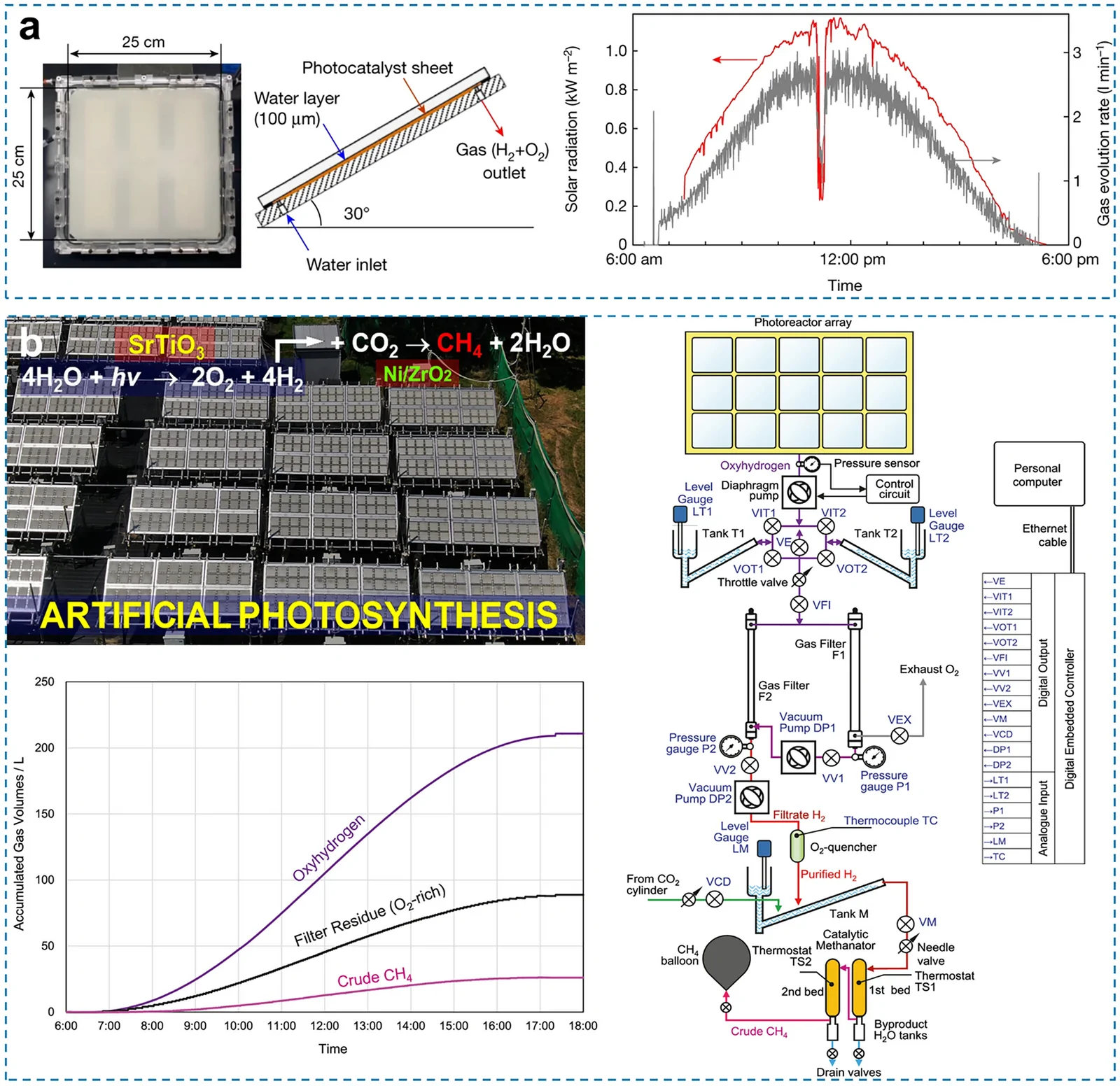
Scale demonstrations of photothermal CO_2_ hydrogenation enabled by solar-to-chemical energy conversion.** a** Panel reactor for solar-driven water splitting. Reproduced with permission from Ref. [199], Copyright 2021 Springer Nature. **b** Artificial photosynthesis system via solar-to-chemical energy conversion. Reproduced with permission from Ref. [200], Copyright 2023 American Chemical Society

Kazunari Domen et al. coupled photocatalytic water splitting with thermocatalytic CO_2_ methanation in an artificial photosynthesis platform (Fig. 20a) [199]. Using a 100 m^2^ panel-type reactor array loaded with SrTiO_3_:Al (Fig. 20b), the photocatalytic unit generated 1770 L of oxyhydrogen in a single day under strong summer sunlight, from which 96.5% pure H_2_ was obtained after gas separation and subsequently used for CO_2_ methanation. During a three-day experiment, the integrated system yielded a total of 43.9 L of crude methane with a purity of 61.2% [200]. In addition, the "kiloton-scale liquid solar fuel synthesis demonstration project" led by Can Li integrated photovoltaic power generation, water electrolysis, and CO_2_ hydrogenation for methanol synthesis. Solar electricity drove large-scale electrolyzers to produce H_2_ at rates above 1000 m^3^ h^−1^, which was then converted with CO_2_ over ZnZrO_x_ solid solution catalysts in an adiabatic fixed-bed reactor. Commissioned in January 2020, the system reached a methanol purity of 99.5 mol% within 3 h.

### Practical Feasibility of Scaled Photothermal CO_2_ Hydrogenation

Although recent scale demonstrations have shown that photothermal CO_2_ hydrogenation can operate under real sunlight, practical deployment requires further evaluation beyond reactor size, product output, or short-term activity. Two issues are particularly important for scaled systems: catalyst durability under long-term photothermal operation and techno-economic feasibility at the process level. The former determines whether activity and selectivity can be maintained under fluctuating light and temperature fields, whereas the latter determines whether solar-driven CO_2_ hydrogenation can compete with established thermocatalytic or fossil-based production routes.

#### Catalyst Feasibility

Catalyst durability is a prerequisite for the practical deployment of photothermal CO_2_ hydrogenation. Compared with conventional thermal catalysis, photothermal catalysis operates under spatially non-uniform light and temperature fields, where localized hot spots and repeated light/dark cycling may accelerate structural evolution of catalysts [130]. This issue becomes more critical for C_2+_ product synthesis, because C–C coupling and chain growth reactions involve multiple surface intermediates and may promote the accumulation of heavier hydrocarbons or carbonaceous species [26, 182]. Therefore, long-term stability should be evaluated not only by maintaining product yield, but also by tracking selectivity evolution, active-site structure, surface temperature distribution, and carbon balance during operation.

The deactivation of photothermal CO_2_ hydrogenation catalysts generally originates from several pathways. First, metal nanoparticles may undergo sintering or aggregation under localized high-temperature conditions, leading to the loss of exposed active sites and weakened metal–support interfaces. For example, post-reaction characterization of plasmonic Pt/TiN showed that Pt species transformed from single atoms/clusters into 1–2 nm nanoparticles under reaction conditions, and this sintering was identified as the reason for the gradual activity decline [168]. Second, active metal or oxide sites may experience oxidation, reduction, or structural reconstruction during redox operation, thereby changing the adsorption and activation behavior of CO_2_ and H_2_. For instance, TiO_x_ overlayers formed on Ni/TiO_2_ after high-temperature reduction were removed under CO_2_ hydrogenation conditions, demonstrating that metal–oxide interfacial structures can dynamically reconstruct during reaction [201]. Third, oxygen vacancies and other surface defects may be gradually consumed or passivated by oxygen-containing intermediates, resulting in decreased CO_2_ activation ability. This possibility is supported by theoretical and experimental studies showing that CO_2_ dissociation at oxygen vacancy sites can insert oxygen into the vacancy and reoxidize the reduced surface. For example, on defective TiO_2_, CO_2_ dissociation at oxygen vacancies leads to CO formation while the reduced surface becomes oxidized, indicating that oxygen vacancies can be consumed during CO_2_ activation [202]. Therefore, when oxygen vacancy regeneration is insufficient, defect-mediated CO_2_ activation may gradually weaken during long-term operation. In addition, photothermal hot spots may induce support phase transformation, pore collapse, or interfacial reconstruction, further reducing catalytic stability. This concern is consistent with general thermal deactivation mechanisms, where high-temperature operation can cause active-phase crystallite growth, loss of support surface area through pore collapse, and transformation of catalytic phases into less active phases.

Improving the durability of photothermal CO_2_ hydrogenation catalysts requires coordinated optimization of catalyst structure, active-site regeneration, and reactor-level heat management. Stable catalyst architectures, such as confined metal nanoparticles, alloy structures, strongly coupled metal–support interfaces, and defect-anchored active sites, can suppress structural degradation under localized photothermal heating [20]. Meanwhile, reducible supports, hydrogen spillover pathways, and redox-cycling treatments can help maintain defect-mediated CO_2_ activation [203]. For catalysts affected by strongly adsorbed intermediates or carbon deposits, controlled regeneration treatments are needed to restore active sites [204]. In addition, uniform irradiation, efficient heat dissipation, and improved mass transport are essential for reducing hot-spot-induced degradation. These strategies should be combined with long-term stability tests and post-reaction characterization to establish reliable durability criteria for practical photothermal CO_2_ hydrogenation.

#### Economic Feasibility

Beyond catalyst durability, the practical deployment of scaled photothermal CO_2_ hydrogenation also depends on whether the overall process can become economically competitive. Economic feasibility should therefore be evaluated from a system-level perspective rather than solely from catalytic activity or reactor productivity. Key cost factors include renewable H_2_ supply, CO_2_ capture and purification, catalyst lifetime, reactor capital investment. These factors are especially important for C_2+_ synthesis, which typically involves higher H_2_ input, more complex reaction networks, exhibits lower single-pass selectivity, and imposes greater downstream separation costs than C_1_ production. Recent techno-economic analysis estimated the levelized costs of photothermal CO_2_ conversion to CO, CH_4_, CH_3_OH, and C_2+_ products to be 723.59, 617.37, 716.91, and 989.07 USD t^−1^, respectively [205]. Since these values remain higher than the corresponding market prices, current photothermal CO_2_ conversion technologies still lack broad cost competitiveness.

Among the system-level cost contributors, green H_2_ plays a central role in determining the economics of CO_2_-derived fuels and chemicals. It has been reported that renewable methanol production using electrolytic H_2_ and flue-gas-captured CO_2_ has a levelized cost of approximately 960 € t^−1^, more than twice the international methanol market price of about 450 € t^−1^. The cost penalty mainly arises from electrolyzer investment and renewable electricity demand [206]. For olefin production through CO_2_ hydrogenation using green H_2_ and CO_2_ captured by direct air capture, the minimum selling price of olefins was estimated to be approximately £3.67 kg^−1^, with electricity cost remaining the dominant cost driver [207]. From the perspective of H_2_ consumption per unit product, methanol production requires approximately 0.188 t H_2_ per ton of CH_3_OH, whereas ethylene production requires approximately 0.429 t H_2_ per ton of C_2_H_4_. Therefore, as the target products extend from C_1_ compounds to C_2+_ products, H_2_ demand and the associated cost pressure will further increase. Reducing the cost of green H_2_, particularly through low-cost renewable electricity, improved electrolyzer utilization, and optimized H_2_ storage and system integration, is therefore essential for the economic viability of photothermal CO_2_ hydrogenation.

The CO_2_ source represents another critical cost variable. Most laboratory and pilot-scale photothermal CO_2_ hydrogenation studies use high-purity CO_2_, whereas industrial deployment would require CO_2_ from point-source capture, biogenic sources, or direct air capture. Among these options, point-source CO_2_ is more attractive because capture cost decreases significantly as CO_2_ concentration increases. According to the International Energy Agency, CO_2_ capture costs are about 15–25 USD t^−1^ for relatively pure or highly concentrated industrial CO_2_ streams, such as those from ethanol production and natural gas processing, but increase to 40–120 USD t^−1^ for more dilute sources, including cement production and coal-fired power generation. By contrast, direct air capture still suffers from substantially higher energy demand and cost because the CO_2_ concentration in air is much lower than that in flue gas or industrial process streams. Therefore, coupling photothermal CO_2_ hydrogenation with highly concentrated or low-cost point-source CO_2_ is likely to be more economically feasible in the near term. Although direct-air-capture-coupled systems are potentially attractive for long-term carbon-neutral or carbon-negative fuel synthesis, their feasibility will depend on substantial reductions in capture cost, regeneration energy demand, and the cost of low-carbon heat or electricity.

From the perspective of scale-up routes, as summarized in Table 7, different pathways exhibit distinct advantages and limitations. Optical concentration-enhanced systems directly enhance photon flux and catalyst temperature, but their productivity is highly sensitive to direct solar irradiance, weather conditions, optical alignment, and tracking efficiency. Moreover, highly concentrated light may increase local product yield but can also waste photons when light penetration, catalyst utilization, and heat distribution are not optimized [205]. Solar-to-thermal systems can to some degree mitigate solar irradiation fluctuations by heat accumulation and are therefore attractive for exothermic reactions such as CO_2_ methanation, as evidenced by recent day-and-night photothermal methanation. However, this approach requires additional costs for solar absorbers, insulation materials, heat transfer modules, and cooling systems [198]. By contrast, solar-to-chemical integrated systems couple photovoltaic water electrolysis or photocatalytic water splitting with CO_2_ hydrogenation, which can partially decouple solar energy harvesting from catalytic conversion and thereby improve operational flexibility. Nevertheless, the economic feasibility of such systems remains highly dependent on the capital cost of water-splitting devices, H_2_ purification and compression costs, and the overall system capacity factor.

**Table 7:** Scale-up demonstrations of solar-driven CO_2_ conversion routes

Route	Catalytic System	Product	Scale	Rate	Stability	Advantages	Disadvantages	Ref.
Optical concentration-enhanced solar energy harvesting	Ni/C-In_2_O_3_	CO	~7 cm^2^	20.96 mmol g_cat_^−1^ h^−1^	2 d	Direct photon flux enhancement Higher catalyst temperature High local productivity Compact optical reactors	Direct irradiance sensitivity Weather dependence Optical alignment demand Limited light penetration Uneven heat distribution	[188]
	Ru/Mo_2_TiC_2_	CO	4.91 cm^2^	3.9 mol g_Ru_^−1^ h^−1^	10 h			[193]
	Ru-Al_2_O_3_−x-L	CH_4_	4 cm^2^	18.00 mmol min^−1^	8 d			[194]
	Ru-TiO_x_	CH_4_	4 cm^2^	~0.95−1.12 mmol g_cat_^−1^ h^−1^	7 d			[195]
	Ru_1_/CeO_2_-AS	CH_4_	~0.785 cm^2^	32.36 mol g_Ru_^−1^ h^−1^	7 d			[208]
	Ru_1_/CeO_2_	CO	~0.785 cm^2^	42.53 mol g_Ru_^−1^ h^−1^	7 d			[208]
	CoFe_2_O_4_ /(Fe_1−x_Co_x_)_5_C_2_	C_2_-C_4_ olefins	706.5 cm^2^	66.1 L m^−2^ day^−1^	1 d			[130]
Solar-to-thermal energy conversion	5%AgK/Fe_3_O_4_	CO	20 m^2^	1925 mmol g^−1^ h^−1^	1 d	Heat accumulation buffering Lower fluctuation sensitivity Suited to exothermic reactions for day-and-night operation	Insulation material cost Heat transfer modules Cooling system demand Thermal management complexity	[64]
	Cu_2_Zn_1_Al_0.5_Ce_5_Zr_0.5_O_x_	CO	0.0396 m^2^	248.5 mmol g^−1^ h^−1^	7 d			[68]
	Ni_1_Ag_0.02_O_1_	CO	9 m^2^	1434 mmol g^−1^ h^−1^	4 d			[197]
	Ni_1_Ce_1_O_3_	CH_4_	N.R.	179.6 m^3^ d^−1^	5 d			[198]
Solar-to-chemical energy conversion	Ni/ZrO_2_	CH_4_	12.19 m^2^	26.9 L d^−1^	3 d	PV–electrolysis coupling Photocatalytic H_2_ coupling Harvesting/conversion decoupling Improved operational flexibility	Water-splitting efficiency H_2_ purification cost Capacity factor dependence Full-system integration cost	[200]

In summary, photothermal CO_2_ hydrogenation offers an attractive strategy for reducing external thermal input by integrating solar energy harvesting and catalytic conversion within a single system. However, its economic advantage over established thermocatalytic processes and fossil-derived chemical production routes remains to be fully demonstrated. Future scale demonstrations should therefore move beyond proof-of-concept validation and provide standardized techno-economic indicators. In the case of C_2+_ products, additional benchmarks, such as C_2+_ carbon selectivity, olefin/paraffin ratio, single-pass carbon efficiency, and separation cost for multi-product streams, should be carefully reported. Establishing such standardized techno-economic benchmarks will be essential for determining whether photothermal CO_2_ hydrogenation can progress from scalable demonstration to economically viable industrial deployment.

## Conclusions and Perspectives

Photothermal CO_2_ hydrogenation to C_2+_ products represents a promising green chemistry approach, offering not only a viable pathway for CO_2_ emission reduction but also significant industrial potential for the synthesis of high-value chemicals. Nevertheless, achieving efficient and selective formation of C_2+_ products remains challenging due to limitations in reactant mass transfer and difficulties in C−C bond formation. This review systematically summarizes recent advances in photothermal CO_2_ hydrogenation to C_2+_ products. The fundamental principles of photothermal catalysis are first discussed from the perspectives of photo-induced, thermal, and synergistic effects. Subsequently, the activation of CO_2_ to C_1_ intermediates and the C−C coupling pathways leading to C_2+_ products are analyzed. Based on these mechanistic insights, we outline a series of catalyst design and modification strategies. To enhance CO_2_ activation toward C_1_ intermediates, we review catalyst engineering approaches that improve photothermal conversion efficiency and promote reactant activation, grounded in core principles of photothermal catalysis. For the C−C coupling of C_1_ intermediates into C_2+_ products, multiple catalyst design strategies are summarized according to different coupling routes. Additionally, recent progress in scaled-up demonstrations is highlighted to support the development of practical, scalable CO_2_ hydrogenation technologies. Overall, this review offers theoretical guidance for the rational design of photothermal catalysts toward efficient and selective CO_2_ conversion to multicarbon products.

Despite notable progress, several critical challenges remain, which should be addressed in future research:

1. At the material level, future progress requires a transition from empirical catalyst optimization to data-driven catalyst construction. Current trial-and-error strategies are inefficient for discovering C_2+_-selective photothermal CO_2_ hydrogenation catalysts as relevant compositional, structural, and interfacial space is extremely broad. Although machine learning has been increasingly used in CO_2_ catalytic conversion, its application to photothermal CO_2_ hydrogenation, especially C_2+_ formation, remains limited. Future studies should establish standardized datasets that integrate catalyst structure, light absorption, and product performance, enabling predictive models for activity and selectivity. Active learning can then identify high-value experiments and update models with informative feedback, while inverse design can generate catalyst candidates that meet predefined targets. In the long term, closed-loop autonomous platforms combining prediction, synthesis, testing, and feedback may accelerate the discovery of efficient and stable C_2+-_selective photothermal catalysts.
2. At the reaction level, future research should shift from activity-oriented CO_2_ conversion to selectivity-oriented C_2+_ synthesis. Current photothermal CO_2_ hydrogenation systems mainly produce C_1_ products, whereas C_2+_ olefin formation is limited by the sluggish kinetics of C–C bond formation and the competing overhydrogenation reaction. As discussed in this review, C–C coupling can proceed through CO*-mediated, CH_x_*-mediated, and CH_3_OH*-mediated pathways. Among them, the CH_3_OH*-mediated route is particularly attractive because it may alleviate the restriction of ASF distribution and improve C_2+_ selectivity through tandem transformation. However, zeolite-based systems have rarely been applied in photothermal catalysis due to their insufficient photothermal response and inefficient charge transport. Future designs should therefore enhance the light-harvesting and charge transfer capabilities of zeolite-based hybrid catalysts. If these limitations can be effectively addressed, a feasible short-term benchmark would be to achieve C_2+_ product selectivity above 70%. In the long term, these improved reaction modules are expected to support stable, pathway-directed C_2+_ synthesis under continuous photothermal operation.
3. At the system level, future research should bridge the gap between short-term photothermal demonstrations and reliable outdoor solar fuel operation. Current photothermal CO_2_ hydrogenation systems can operate under natural sunlight, but their stability remains limited by fluctuating solar irradiation, which induces variations in reactor temperature, reaction rate, H_2_ demand, and product distribution. Future systems should therefore integrate solar energy collection, thermal buffering, green H_2_ supply, CO_2_ conversion, and product separation into an adaptive platform. In such systems, H_2_ supply should be dynamically matched with real-time temperature and catalytic consumption to improve resource utilization. Machine learning-driven multi-objective optimization can further identify stable operating windows by balancing C_2+_ productivity, product selectivity, solar-to-chemical efficiency, H_2_ utilization, and operational stability. Once such optimized operating windows are established, the next practical milestone should be stable outdoor operation over hundreds of hours with quantified solar-to-chemical efficiency.
4. At the industrial level, the key challenge is to translate scalable photothermal CO_2_ hydrogenation from demonstration systems into economically meaningful processes. Although recent scale-up studies suggest practical potential, larger reactor size or longer operation time alone cannot establish industrial feasibility. The total process cost is strongly affected by green H_2_ supply, CO_2_ capture and purification, reactor construction, catalyst durability, and downstream separation. This challenge is more pronounced for C_2+_ products, where higher H_2_ demand and multi-product separation increase both energy input and operating cost. Therefore, future demonstrations should be guided by techno-economic analysis and life-cycle assessment at the design stage, with levelized product cost and carbon intensity reported together with catalytic performance. In the near term, deployment should prioritize low-cost point-source CO_2_ and renewable H_2_ to establish economically favorable scenarios. In the long term, cost parity with fossil-derived products and verified life-cycle carbon reduction should become the criteria for industrial relevance.
